# Destabilization of macrophage migration inhibitory factor by 4‐IPP reduces NF‐κB/P‐TEFb complex‐mediated c‐Myb transcription to suppress osteosarcoma tumourigenesis

**DOI:** 10.1002/ctm2.652

**Published:** 2022-01-20

**Authors:** Lin Zheng, Zhenhua Feng, Siyue Tao, Jiawei Gao, Ye Lin, Xiaoan Wei, Bingjie Zheng, Bao Huang, Zeyu Zheng, Xuyang Zhang, Junhui Liu, Zhi Shan, Yilei Chen, Jian Chen, Fengdong Zhao

**Affiliations:** ^1^ Department of Orthopaedic Surgery Sir Run Run Shaw Hospital, Medical College of Zhejiang University & Key Laboratory of Musculoskeletal System Degeneration and Regeneration Translational Research of Zhejiang Province 3 East Qingchun Road Hangzhou Zhejiang Province 310016 China

**Keywords:** 4‐IPP, CDK9, c‐Myb, MIF, osteosarcoma, STUB1

## Abstract

**Background:**

As an inflammatory factor and oncogenic driver protein, the pleiotropic cytokine macrophage migration inhibitory factor (MIF) plays a crucial role in the osteosarcoma microenvironment. Although 4‐iodo‐6‐phenylpyrimidine (4‐IPP) can inactivate MIF biological functions, its anti‐osteosarcoma effect and molecular mechanisms have not been investigated. In this study, we identified the MIF inhibitor 4‐IPP as a specific double‐effector drug for osteosarcoma with both anti‐tumour and anti‐osteoclastogenic functions.

**Methods:**

The anti‐cancer effects of 4‐IPP were evaluated by wound healing assay, cell cycle analysis, colony formation assay, CCK‐8 assay, apoptosis analysis, and Transwell migration/invasion assays. Through the application of a luciferase reporter, chromatin immunoprecipitation assays, and immunofluorescence and coimmunoprecipitation analyses, the transcriptional regulation of the NF‐κB/P‐TEFb complex on c‐Myb‐ and STUB1‐mediated proteasome‐dependent MIF protein degradation was confirmed. The effect of 4‐IPP on tumour growth and metastasis was assessed using an HOS‐derived tail vein metastasis model and subcutaneous and orthotopic xenograft tumour models.

**Results:**

In vitro, 4‐IPP significantly reduced the proliferation and metastasis of osteosarcoma cells by suppressing the NF‐κB pathway. 4‐IPP hindered the binding between MIF and CD74 as well as p65. Moreover, 4‐IPP inhibited MIF to interrupt the formation of downstream NF‐κB/P‐TEFb complexes, leading to the down‐regulation of c‐Myb transcription. Interestingly, the implementation of 4‐IPP can mediate small molecule‐induced MIF protein proteasomal degradation via the STUB1 E3 ligand. However, 4‐IPP still interrupted MIF‐mediated communication between osteosarcoma cells and osteoclasts, thus promoting osteoclastogenesis. Remarkably, 4‐IPP strongly reduced HOS‐derived xenograft osteosarcoma tumourigenesis and metastasis in an in vivo mouse model.

**Conclusions:**

Our findings demonstrate that the small molecule 4‐IPP targeting the MIF protein exerts an anti‐osteosarcoma effect by simultaneously inactivating the biological functions of MIF and promoting its proteasomal degradation. Direct destabilization of the MIF protein with 4‐IPP may be a promising therapeutic strategy for treating osteosarcoma.

## Graphical abstract

1. MIF inhibitor 4‐IPP reduced the proliferation and metastasis of osteosarcoma cells by suppressing the NF‐κB pathway.

2. MIF inhibitor 4‐IPP interrupted the MIF/CD74‐induced NF‐κB/P‐TEFb complex‐mediated c‐Myb transcription in osteosarcoma.

3. MIF inhibitor 4‐IPP mediated MIF protein proteasomal degradation via the STUB1 E3 ligase.

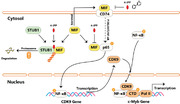

## 1 INTRODUCTION

Osteosarcoma is a common bone tumour caused by mesenchymal cells. Among all cancers affecting children and adolescents, osteosarcoma has the highest mortality rate.^1^, ^2^ Even with the variety of applied treatment methods, including chemotherapy, the 5‐year survival rate is still lower than 70 per cent because of tumour recurrence related to metastasis and drug resistance.^3^ In addition, effective therapeutic targets or diagnostic markers for osteosarcoma have not been identified; thus, the therapeutic effect is difficult to improve. Therefore, better strategies and new treatments are urgently needed to address drug resistance and distant metastasis to improve the osteosarcoma prognosis.

As a pleiotropic cytokine, macrophage migration inhibitory factor (MIF) is expressed in immune cells, endocrine cells and epithelial cells, among others.^4^ As reported recently, MIF also contributes to tumour progression and malignant transformation, such as in lung cancer,^5^ hepatocellular carcinoma,^6^ melanoma^7^ and osteosarcoma.^8^ Inhibition of MIF activity in animal models of urogenital cancer produced an anti‐tumour effect by downregulating cancer‐associated signaling pathways, including p53, PKB and ERK pathways.^9^ In human myeloma, knocking out MIF is able to overcome resistance to proteasome inhibitors, and MIF has been identified as a biomarker and therapeutic target.^10^ In osteosarcoma,^8^ MIF activates the MAPK pathway to promote lung metastasis as well as tumour growth, indicating that immunotherapy targeting MIF may represent a viable and promising treatment for osteosarcoma. Our previous study presented the pharmacological targeting of MIF by 4‐iodo‐6‐phenylpyrimidine (4‐IPP) for treating osteolytic bone disorders.^11^ The effect of MIF inhibitors on osteosarcoma has not been clarified, and an inhibitor that targets MIF to treat osteosarcoma remains to be identified.

Herein, we identified 4‐IPP as a specific double‐effector drug for osteosarcoma with both anti‐tumour and anti‐osteoclastogenic functions. Furthermore, treatment with 4‐IPP could effectively inhibit osteosarcoma cell metastasis and proliferation through transcriptional regulation of c‐Myb by the NF‐κB/P‐TEFb complex. Importantly, we found that 4‐IPP could mediate the degradation of the MIF protein via the STUB1 E3 ligand. These results indicate that 4‐IPP can be used in a novel treatment strategy for osteosarcoma.

## 2 MATERIALS AND METHODS

### 2.1 Animal studies

Approval was obtained from the ethics committee of Zhejiang University for the animal experiments and human osteosarcoma samples, which followed the standards of the Guide for the Care and Use of Laboratory Animals from the National Institutes of Health.

### 2.2 Cell culture and transfection

Human osteoblast hFOB1.19 (American Type Culture Collection [ATCC]: CRL‐11372), MG‐63 (ATCC: CRL‐1427TM), HOS (ATCC: CRL‐1543), 143B (ATCC: CRL‐8303), SJSA‐1 (ATCC: CRL‐2098) and U2OS (ATCC: HTB96TM) cell lines were obtained from the (ATCC, Manassas, VA, USA). hFOB 1.19 cells were cultured in Dulbecco's modified Eagle's medium/nutrient mixture F‐12 (DMEM/F‐12) and 10% fetal bovine serum (Gibco, Grand Island, NY, USA), and the other cell lines were cultured in DMEM and 10% fetal bovine serum (Gibco), at 37°C in 5% CO_2_. Plasmids were transfected using Lipofectamine 3000 (Life Technologies, Waltham, Massachusetts, USA) following the manufacturer's instructions.

### 2.3 Drug affinity responsive target stability

M‐PER (Thermo Fisher Scientific, Waltham, Massachusetts, USA) with phosphatase inhibitor and protease inhibitor cocktails (Roche, Basel, Switzerland) was applied to lyse the cells, and then target verification by drug affinity responsive target stability (DARTS) was performed.^12^, ^13^ The cell lysates were added to TNC buffer (10 mM CaCl2, 50 mM NaCl and 50 mM Tris‐HCl pH 8.0) and incubated with either 4‐IPP or the vehicle (DMSO) on ice for 1 h and then at room temperature for another 20 min. Then, the cell lysates were digested at room temperature for 20 min by Pronase (Roche, Basel, Switzerland), which was stopped using SDS loading buffer, after which a heat treatment was applied at 70 °C for 10 min. SDS‐PAGE and western blotting were then performed for the indicated proteins.

### 2.4 Histopathology and immunohistochemistry

To prepare the animal tissue samples, we embedded the formalin‐fixed tissue samples in paraffin, cut them into 4 μm sections and placed them on slides. For immunohistochemical staining, the slides were dewaxed in xylene and rehydrated with graded alcohol. Then, the slides were incubated in 3% hydrogen peroxide to impede endogenous peroxidase activity. The slides were boiled for 30 min in 10 mM sodium citrate (pH 6.0) for antigen retrieval, blocked with 5% normal goat serum for 15 min and then incubated with the indicated antibodies overnight at 4°C in a humid room. The next day, the slides were incubated with the secondary antibody for 1 h at room temperature after the PBS washing process. A metal enhanced DAB substrate kit (Solarbio Life Sciences, Peking, China) was used to detect immunoreactivity. The antibody staining intensity in tissue sections was quantitatively assessed by an IHC Profiler^14^ using ImageJ (an open‐source plugin for quantitatively evaluating and automatically scoring immunohistochemistry images of human tissue samples). The IHC profiler uses the average gray value of positive cells (staining intensity) and the percentage of positive area (stained area) as IHC measurement indicators and ultimately gives four scores: high positive (3+), positive (2+), low positive (1+) and negative (0).

### 2.5 Virtual docking assay

The crystal structures of the MIF1 trimer and STUB1 dimer were provided by the RCSB Protein Database Bank, with PDB code 3WNS (chains A, B and C)^15^ and PDB code: 6NSV (chains A and B),^16^ respectively. For the docking experiment, we analysed and prepared the biopolymer structure, which was supplemented by hydrogen atoms with H‐bond orientation. All water molecules and ligands were removed.

### 2.6 Statistical analysis

Prism 8 (GraphPad Software, USA) was adopted for statistical analysis. The data were analysed using either one‐way ANOVA when necessary or Student's *t*‐test. The results were expressed as the mean ± SD. Statistically significant differences were identified as a *p*‐value of less than 0.05.

Additional experimental procedures used in this study can be found in Supplementary Materials and Methods online.

## 3 RESULTS

### 3.1 The MIF inhibitor 4‐IPP suppressed the proliferation of osteosarcoma cells by promoting MIF protein degradation

A previous study^8^ reported that MIF expression is up‐regulated in osteosarcoma patient tissues. Here, MIF expression in osteosarcoma samples at different stages was tested. We collected 40 samples of osteosarcoma at different stages and performed immunohistochemical detection of MIF. As shown in Figure S1A‐E, except for one case of stage I, all other samples showed at least a low MIF expression level, with the only case of stage IV showing a ‘high positive (+++)' level. The MIF level showed an increasing trend as the osteosarcoma stage increased (for patient information, refer to Table S1). Samples at stage II showed a higher proportion of ‘high positive (+++)' and a lower proportion of ‘negative' results, and the only stage IV patient showed a ‘high positive (+++)' level.

**FIGURE 1:**
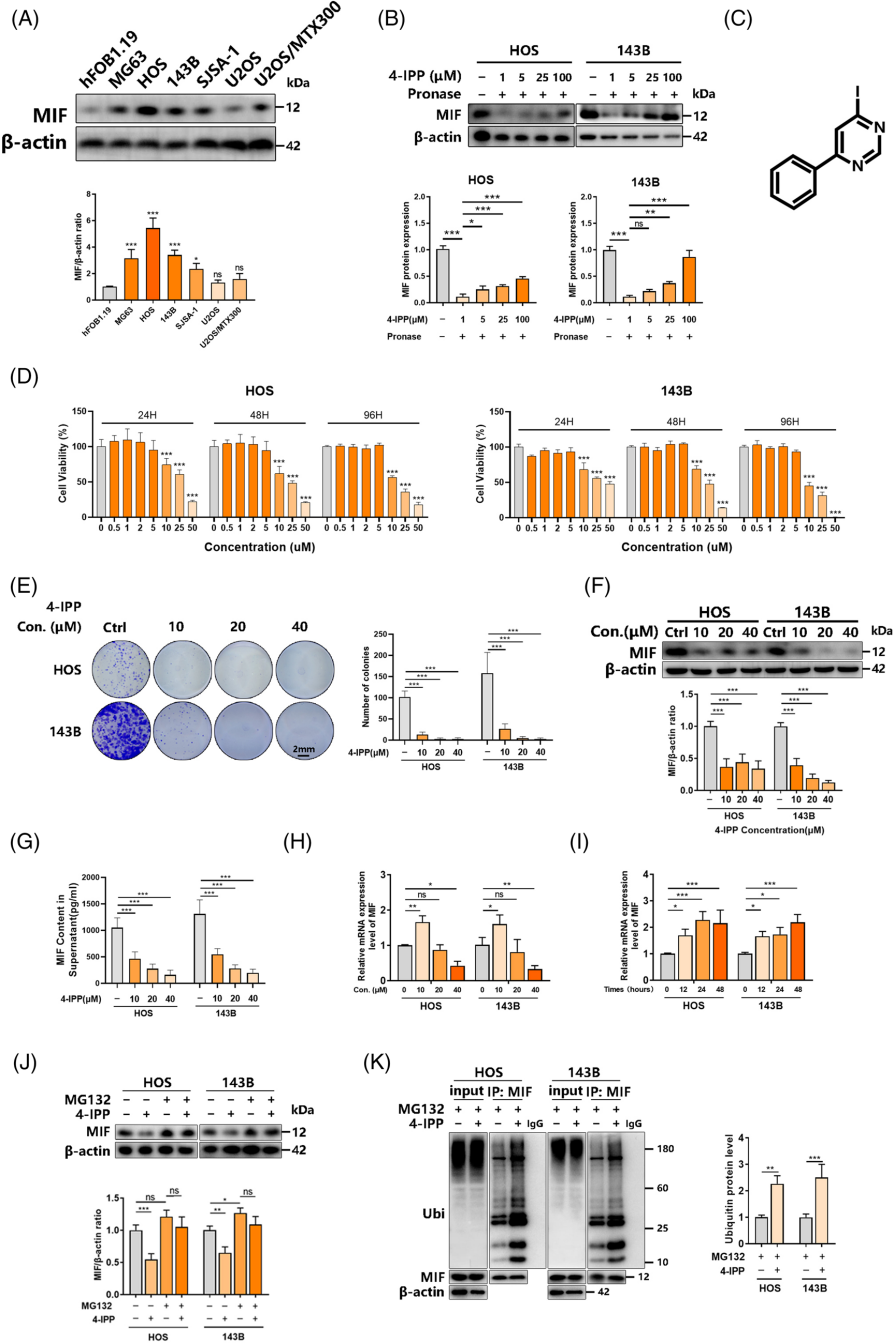
The migration inhibitory factor (MIF) inhibitor 4‐iodo‐6‐phenylpyrimidine (4‐IPP) suppresses the proliferation of osteosarcoma cells by promoting MIF protein degradation. (A) MIF expression in various human osteosarcoma cell lines (U2OS, MG63, SJSA‐1, HOS, 143B, and U2OS/MTX300) and normal cells (osteoblast hFOB1.19) was detected by western blotting (top) and quantification of the indicated protein levels (bottom), and the significance of each group is compared to hFOB1.19 group. (B) HOS/143B cells were incubated with different concentrations of 4‐IPP and Pronase, and DARTS experiments were performed. The binding of 4‐IPP and MIF protein was detected via western blotting (left), and the indicated protein levels were quantified (right). (C) Molecular formula of the 4‐IPP inhibitor. (D) Varying concentrations of 4‐IPP were applied to HOS and 143B cells, and CCK‐8 assays were performed to test viability at 24, 48, and 96 h. (E) The colony formation ability of HOS/143B cells (left) was hindered by 4‐IPP, and the number of colonies was quantified (right). (F) 4‐IPP inhibited MIF expression in HOS/143B cells, as shown by western blotting (top) and quantification of the indicated protein levels (bottom). (G) Effects of 4‐IPP at different concentrations on the level of MIF content in supernatant of HOS/143B cell cultures. (H) Effects of 4‐IPP at different concentrations on the level of MIF mRNA in HOS/143B cells. (I) Changes in MIF mRNA levels in HOS/143B cells treated with 5 μM 4‐IPP for different periods. (J) 4‐IPP (10 μM) or MG‐132 (10 μM) was used to treat HOS/143B cells, which was followed by the lysing process. Western blotting (left) and quantification of the indicated protein levels (right) were performed to detect the interaction of MIF with ubiquitin. (K) HOS/143B cells were treated with MG‐132 (10 μM) supplemented with or without 4‐IPP (10 μM), which was followed by lysis, IgG as a negative control. Coimmunoprecipitation and western blotting (left) and quantification of the indicated protein levels (right) were performed to detect the interaction of MIF with ubiquitin. (Data were obtained from triplicate experiments and are expressed as the mean ± SD; *p < 0.05, **p < 0.01, ***p < 0.001 for a comparison with the control group or as indicated)

Furthermore, we tested the expression of MIF in various osteosarcoma cell lines. In terms of the MIF content, multiple osteosarcoma cell lines (MG63, HOS, 143B, SJSA‐1, U2OS and U2OS/MTX300) showed much higher values than the hFOB1.19 cell line. The HOS and 143B cell lines presented high expression of the MIF protein and hence were chosen for subsequent experiments (Figure 1A). MIF also has two homologous proteins with similar functions, D‐dopachrome tautomerase (DDT) and DDT like (DDTL). We tested the mRNA expression of MIF, DDT and DDTL in HOS/143B cells. The results showed that the mRNA expression of MIF is much higher than that of DDT and DDTL (Figure S2A). As an inhibitor of MIF, 4‐IPP^17^ acts as a suicide substrate to inactivate MIF catalytic and biological functions (Figure 1C). DARTS was used to clear the interaction between 4‐IPP and MIF protein in osteosarcoma cells. It relies on the protection against proteolysis conferred on the target protein by interaction with a small molecule. We demonstrated the use of DARTS^12^ to identify known small‐molecule‐protein interactions. As the concentration of 4‐IPP increased, the combination of 4‐IPP and MIF protein gradually increased (Figure 1B). To examine the IC50 value of 4‐IPP for the growth of human osteosarcoma, varying concentrations of 4‐IPP were used to treat HOS/143B cells for 24/48/96 h. According to the CCK‐8 results, HOS or 143B cell growth was reduced by 50% at 26.79 μM or 37.64 μM for 24 h, 20.17 μM or 20.86 μM for 48 h, and 16.34 μM or 11.74 μM for 96 h, respectively (Figure 1D, Figure S2B). Thus, 4‐IPP concentrations of 10, 20 and 40 μM were then adopted. A colony formation assay showed that 4‐IPP has a significant role in hindering the colony‐forming ability of osteosarcoma cells (Figure 1E). Meanwhile, the inhibitory effects of 4‐IPP on HOS/143B cells were also identified by a soft agar assay (Figure S2C). According to a flow cytometry analysis of the cell cycle, 4‐IPP played a noticeable role in inducing G2‐phase cell cycle arrest and reducing the G1‐phase fraction in a dose‐dependent manner (Figure S2D). Taken together, these results indicate that 4‐IPP could be a potential agent for suppressing sarcoma cell proliferation.

Given the anti‐growth effect of 4‐IPP, we treated HOS/143B cells with 4‐IPP and found that the expression of MIF protein decreased (Figure 1F). The trend of the MIF content in the supernatant was consistent with that of protein expression (Figure 1G). Interestingly, the MIF mRNA level in HOS/143B cells increased with 4‐IPP at 10 μM but decreased at higher concentrations (Figure 1H). Based on inconsistent data exhibited by 10 μM 4‐IPP, we suspected that the low concentration of 4‐IPP may have a negative feedback effect on the MIF mRNA level. We tested the effect of concentrations below the inhibitory concentration on the MIF mRNA level. At a concentration of 5 μM, the MIF mRNA level in HOS/143B cells gradually increased with increasing time (0, 12, 24, and 48 h) (Figure 1I). According to this contradictory result, we first assumed that 4‐IPP may regulate protein degradation to influence the MIF protein content. Therefore, the proteasome inhibitor MG132 was used to identify the influence of protein degradation on the effects of 4‐IPP treatment on osteosarcoma. The MIF protein content was obviously reduced by the 4‐IPP treatment but was relatively improved under treatment with both 4‐IPP and MG132 (Figure 1J). We further tested the ubiquitination of MIF treated with 4‐IPP in osteosarcoma. As shown in Figure 1K, 4‐IPP significantly increased the ubiquitinated form of MIF. These findings reveal the potential effect of 4‐IPP on transcriptional regulation and protein degradation.

### 3.2 The MIF inhibitor 4‐IPP impacts osteosarcoma apoptosis, migration and invasion

**FIGURE 2:**
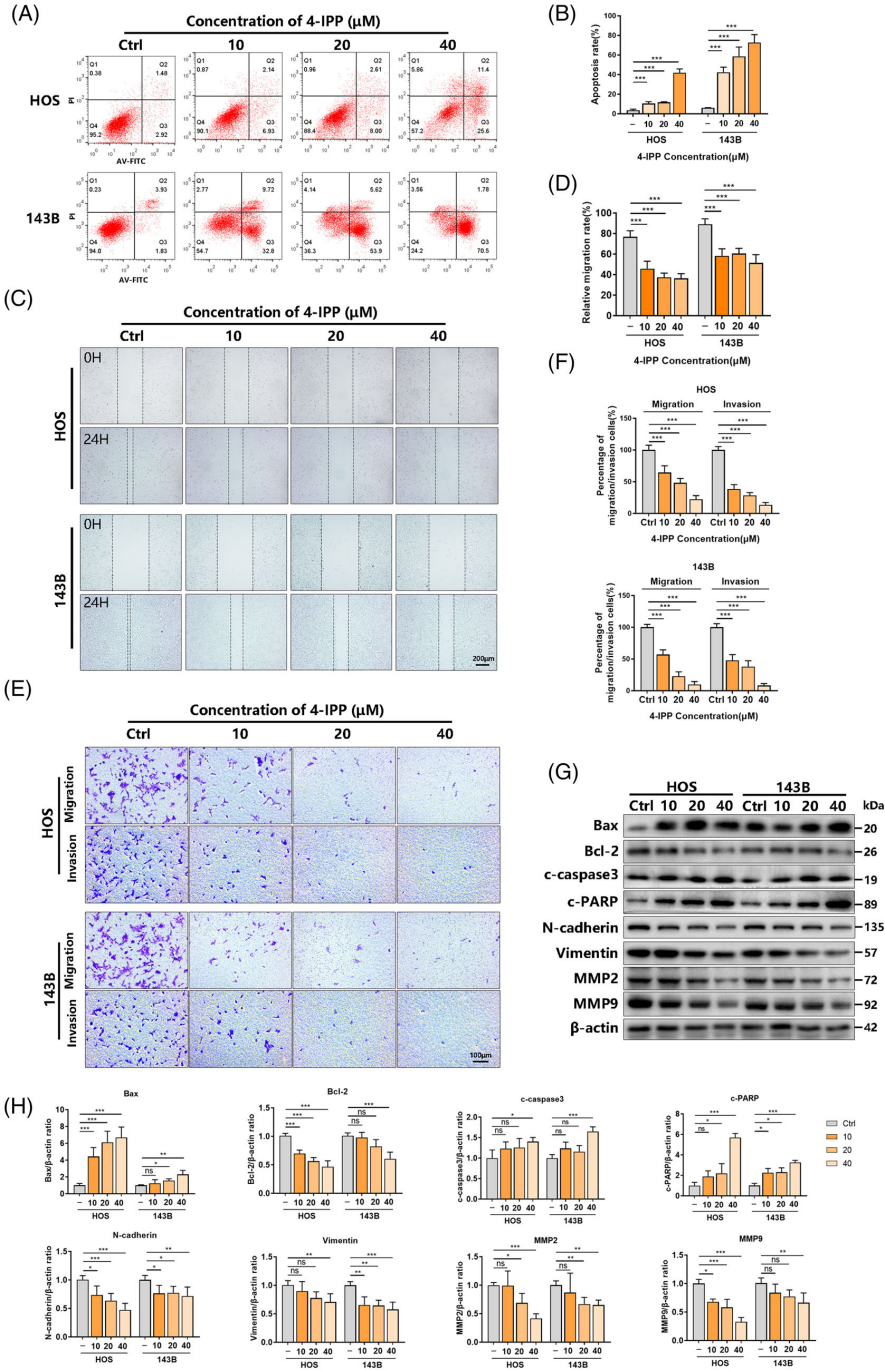
Migration inhibitory factor (MIF) inhibitor 4‐iodo‐6‐phenylpyrimidine (4‐IPP) influences the apoptosis, migration, and invasion abilities of osteosarcoma. (A) Varying concentrations of 4‐IPP were used to treat HOS and 143B cells for 2 days, followed by the staining process by PI and V‐FITC. To determine the percentage of apoptotic cells, flow cytometry analysis was performed. (B) For each treatment, quantification was performed to determine the percentage of apoptotic cells. (C) Scratch wounding by pipette tips was performed, and the HOS and 143B cells were cultured with varying concentrations of 4‐IPP in the medium for 1 day. (D) Quantification was performed for the number of migrated or invaded cells. (E) Cell migration and invasion were measured in Transwell chambers (Matrigel‐coated Transwell chambers for invasion) for 24 h. (F) Quantification was performed on the migration and invasion abilities of HOS/143B cells. (G) Western blotting was performed to assess the protein expression of Bax, Bcl‐2, cleaved caspase 3, cleaved PARP, N‐cadherin, vimentin, MMP2 and MMP9 in HOS/2143B cells treated with varying concentrations of 4‐IPP. (H) ImageJ‐based quantification and normalization of the gray levels of the above proteins to that of β‐actin. (Data were obtained from triplicate experiments and are expressed as the mean ± SD; *p < 0.05, **p < 0.01, ***p < 0.001 for a comparison with the control group or as indicated)

The observations showing the inhibitory effect of 4‐IPP on HOS/143B cell proliferation prompted further investigation into its potential role. The influence of 4‐IPP on cell apoptosis was explored through flow cytometry. Figure 2A,B shows that 4‐IPP promotes the apoptosis of HOS/143B cells in a concentration‐dependent manner. After treatment with 4‐IPP, the scratches in the wound healing assay presented a slow healing rate, indicating that 4‐IPP hindered HOS/143B cell migration (Figure 2C,D). The migration and invasion capacities of HOS and 143B cells were also impeded, as revealed by Transwell migration and invasion assay (Figure 2E,F). The apoptosis marker Bcl‐2 was significantly down‐regulated, while Bax, cleaved caspase 3 and cleaved PARP were markedly up‐regulated after 4‐IPP treatment (Figure 2G,H). The EMT markers N‐cadherin, vimentin, MMP2 and MMP9 were significantly down‐regulated after treatment (Figure 2G,H). After treatment with 4‐IPP, MMP activity in conditioned medium (CM) was detected by gelatin zymography. 4‐IPP treatment obviously decreased the MMP2/MMP9 activity of HOS and 143B cells (Figure S2E).

**FIGURE 3:**
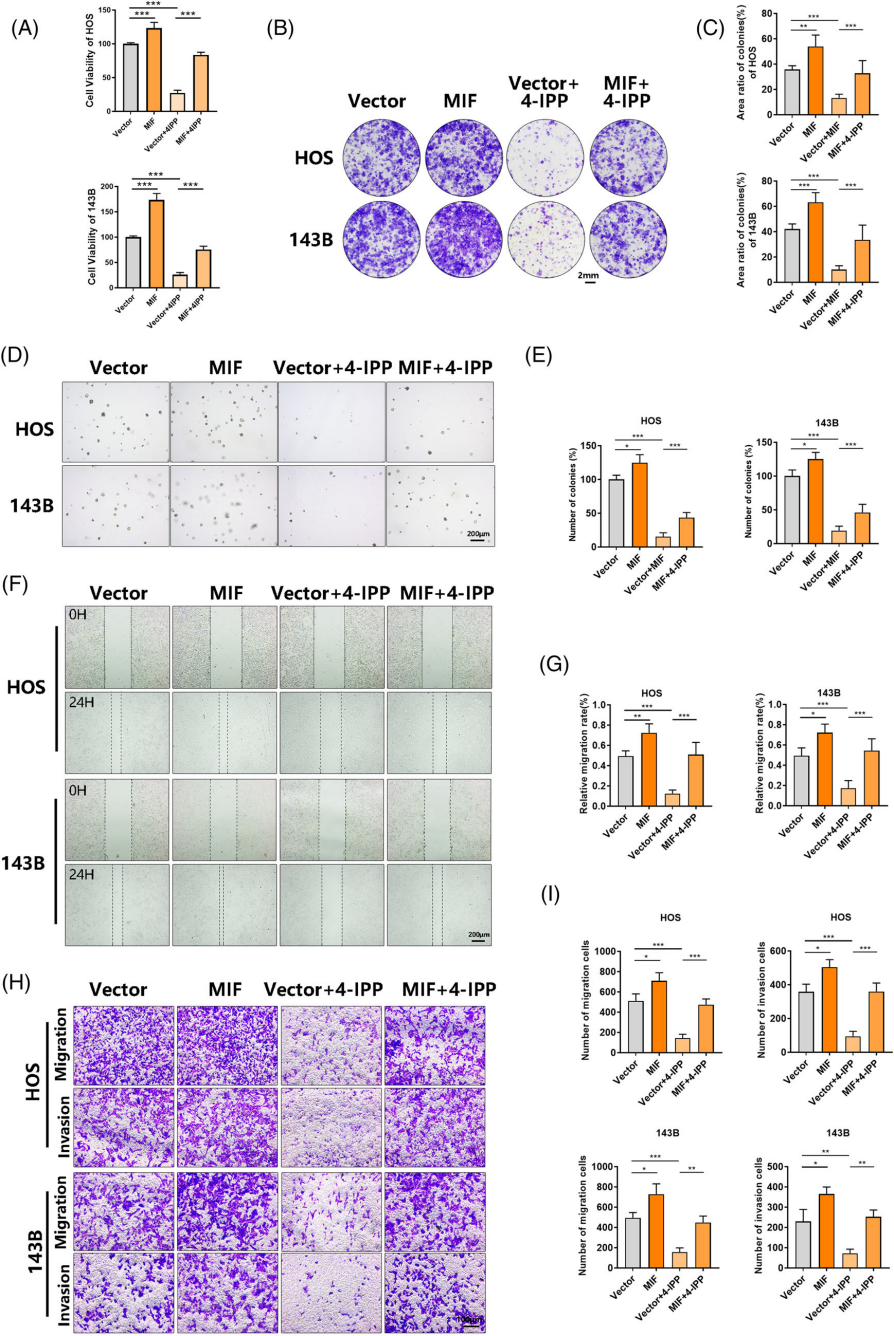
Migration inhibitory factor (MIF) overexpression rescues the inhibition of 4‐iodo‐6‐phenylpyrimidine (4‐IPP) in osteosarcoma. (A) After stable transfection with the vector plasmid or MIF plasmid, HOS and 143B cells were treated with 4‐IPP for 48 h, and viability was tested by CCK‐8 assay. (B) Effect of treatment with 4‐IPP and overexpression of MIF on the colony‐forming ability of osteosarcoma cells based on colony formation assays. (C) Quantification of the number of colonies. (D) Effect of treatment with 4‐IPP and overexpression of MIF on the colony‐forming ability of osteosarcoma cells based on representative images of the soft agar colony formation assay (scale bars, 100 μm). (E) Quantification of the number of colonies. (F) The wound healing assay demonstrated the reversion of migration ability by MIF overexpression. Representative images are shown. (G) The migration ability of osteosarcoma cells was quantified. (H) Transwell migration and Matrigel invasion assays were performed to evaluate cell migration and invasion. Scale bars, 100 μm. (I) The number of migrated or invaded cells was quantified. (Data were obtained from triplicate experiments and are expressed as the mean ± SD; *p < 0.05, **p < 0.01, ***p < 0.001 for a comparison with the control group or as indicated)

We performed further experiments to explore whether 4‐IPP plays an anti‐tumour role in osteosarcoma by participating in MIF rescue. A plasmid of the GV492 vector that can overexpress MIF was constructed, and it showed considerable overexpression efficiency in HOS/143B cells (Figure S3A). Stable transfection of HOS and 143B cells was achieved by an MIF overexpression plasmid. CCK‐8 (Figure 3A), colony formation (Figure 3B,C) and soft agar colony formation (Figure 3D,E) assays were performed to evaluate the proliferation ability of osteosarcoma cells. The exogenous up‐regulation of MIF expression was found to block the 4‐IPP treatment‐induced impairment of proliferation ability. Then, wound‐healing assays (Figure 3F,G) and Transwell migration and Matrigel invasion assays (Figure 3H,I) were performed to evaluate the metastatic ability of osteosarcoma. The exogenous up‐regulation of MIF expression was found to reverse the 4‐IPP treatment‐induced impairment of migration and invasion. Western blotting was performed to assess the protein expression of Bax, Bcl‐2, N‐cadherin and Vimentin in HOS/143B cells. The exogenous up‐regulation of MIF expression was found to reverse the 4‐IPP treatment‐induced change in these proteins (Figure S3B,C). The supernatant was subjected to a gelatin zymography assay to detect the activity of MMP2/MMP9 (Figure S3D) and got consistent results. These findings suggest that MIF overexpression could abolish the anti‐tumour effects of 4‐IPP treatment.

Furthermore, a lentiCRISPRv2 vector was used to knock out MIF gene in HOS/143B cells and showed sufficient knockout efficiency (Figure S4A). The knockout of MIF was found to abolish the 4‐IPP treatment‐induced impairment of proliferation ability according to the CCK‐8 assay (Figure S4B). Western blotting was performed to assess the protein expression of Bax, Bcl‐2, N‐cadherin and Vimentin in HOS/143B. The knockout of MIF expression was found to abolish the 4‐IPP treatment‐induced change in the expression of these proteins (Figure S4C,D). In the migration assay, Matrigel invasion assay and gelatin zymography assay, 4‐IPP also lost its effect after MIF was knocked out (Figure S4E,F). We also tested the effect of 4‐IPP on MIF‐related receptor mRNA and protein expression level. The results suggested that 4‐IPP treatment had no effect on MIF receptor expression (Figure S4G,H).

### 3.3 4‐IPP suppresses MIF/CD74‐induced NF‐κB activation

**FIGURE 4:**
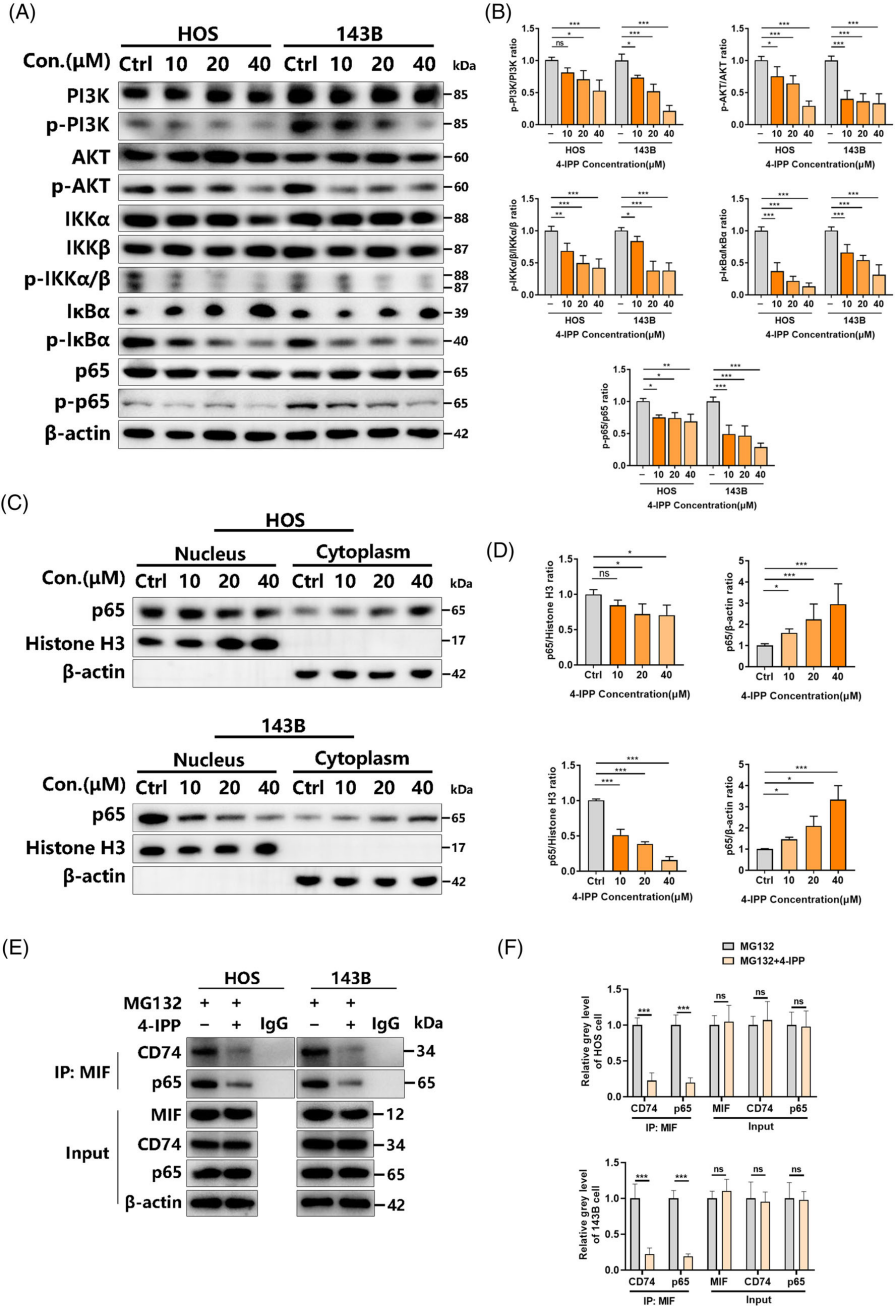
The migration inhibitory factor (MIF) inhibitor 4‐iodo‐6‐phenylpyrimidine (4‐IPP) suppresses the PI3K/AKT/NF‐κB pathway. (A) HOS and 143B cells received 12 h of 4‐IPP treatment, which was followed by western blotting‐based analysis of the cell lysates. (B) The gray levels of phosphorylated p65, PI3K, IκBα, AKT, and IKKα/β were quantified and normalized to the total level by ImageJ. (C) HOS/143B cells received 12 h of 4‐IPP treatment, which was followed by western blotting‐based analysis of the nucleoproteins and cytoplasmic proteins. (D) Quantification and normalization of the gray levels of the indicated protein were performed relative to that of β‐actin using ImageJ. (E) HOS/143B cells were stimulated with 40 μM of 4‐IPP for 12 h, and total cellular proteins extracted were subjected to immunoprecipitation using specific antibodies against MIF. Immunoprecipitates were then subjected to western blot analyses using specific antibodies against p65 and CD74. (F) Quantification of the gray levels of the indicated protein and normalization to that of β‐actin were performed using ImageJ. (Data were obtained from triplicate experiments and are expressed as the mean ± SD; *p < 0.05, **p < 0.01, ***p < 0.001 for a comparison with the control group or as indicated)

Previous studies^8^, ^11^, ^18^ have shown that MIF can promote the PI3K and MAPK pathways, while 4‐IPP has a significant inhibitory effect on the NF‐κB pathway. Thus, we explored how 4‐IPP affected the MAPK and NF‐κB pathways in osteosarcoma cells. Under 4‐IPP treatment with a concentration gradient (0/10/20/40 μM), the phosphorylation of IκBα, IKKα/β, AKT, PI3K and p65 was significantly reduced, the total IKKα/β, AKT, PI3K and p65 were not affected, and the total IκBα was increased, as shown in Figure 4A,B. A similar result was obtained under time gradient conditions (0/3/6/12 h) (Figure S5A,B). The nuclear translocation of p65 was used to induce NF‐κB activation, which inhibited subsequent treatment with 4‐IPP in HOS/143B cells (Figure 4C,D). For the MAPK pathway, treatment with 4‐IPP for different times (0/3/6/12 h) indicated that 4‐IPP could obviously inhibit the phosphorylation of JNK and ERK and slightly inhibit the phosphorylation of p38, and it did not affect the total JNK, ERK and p38 (Figure S5C,D). After MIF overexpression in HOS/143B cells, the exogenous up‐regulation of MIF expression reversed the 4‐IPP treatment‐induced inhibition of p‐AKT and p‐p65 (Figure S5E,F). After MIF knockout in HOS/143B cells, 4‐IPP also lost its effect on p‐AKT and p‐p65 (Figure S5G,H). Previous studies indicated that the interaction of MIF/CD74 and MIF/p65 may be related to the activation of NF‐κB mediated by MIF.^11^, ^17^, ^19^, ^20^ The results of co‐immunoprecipitation experiments show that 4‐IPP can block the binding of MIF and p65 or CD74 (Figure 4E,F). Given that the downstream pathway of MIF/CD74 in osteosarcoma is unknown, we used rhMIF and CD74 neutralizing antibodies to verify the effect of MIF/CD74 on NF‐κB pathway. Different concentrations of rhMIF (1/10/100 ng/ml) will activate the phosphorylation of p65 in HOS/143B cells (Figure S6A). The 4‐IPP and CD74 neutralizing antibodies can antagonize the activation of NF‐κB by rhMIF (Figure S6B‐D). This indicates that extracellular MIF can activate the NF‐κB pathway through CD74 in HOS/143B cells. These findings suggest that 4‐IPP can abolish MIF/CD74‐induced NF‐κB activation.

### 3.4 The MIF inhibitor 4‐IPP inhibits c‐Myb expression by suppressing formation of the NF‐κB/CDK9 transcriptional complex

**FIGURE 5:**
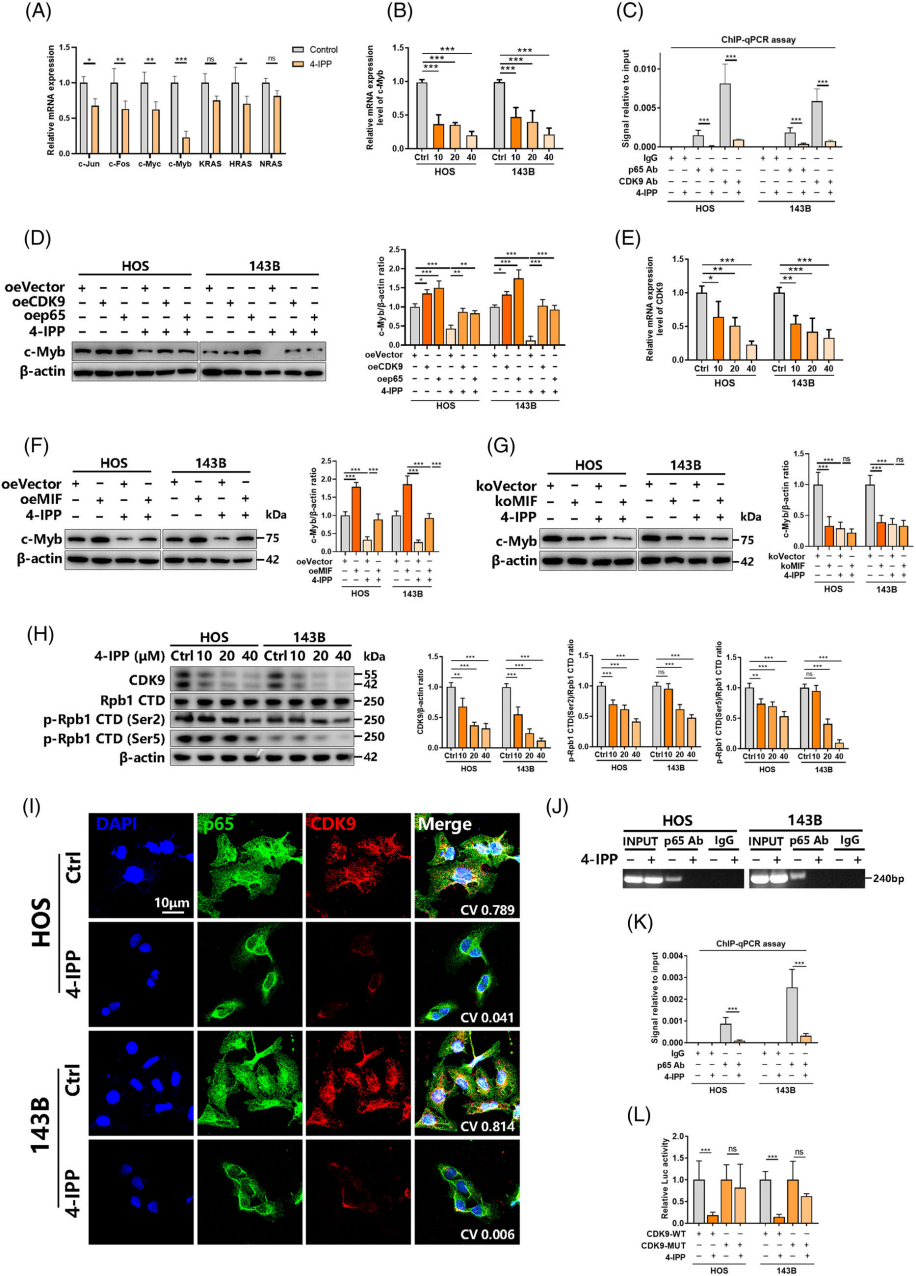
The migration inhibitory factor (MIF) inhibits 4‐iodo‐6‐phenylpyrimidine (4‐IPP) inhibited c‐Myb expression by suppressing the formation of the NF‐κB/CDK9 transcriptional complex. (A) HOS cells were treated with 4‐IPP for 48 h, followed by qRT‐PCR‐based detection of different proto‐oncogenes. (B) HOS and 143B cells received 48 h of 4‐IPP treatment, which was followed by qRT‐PCR‐based detection of the mRNA level of c‐Myb. (C) Correlation between the transcription factor p65 and CDK9 protein complex levels and the c‐Myb promoter regions in HOS and 143B cells was analysed by ChIP assay. (D) HOS/143B cells were transfected with the CDK9/p65 overexpression plasmid (pCMV6‐entry as vector). Cells were lysed, and proteins were detected by western blotting (left). CDK9 and c‐Myb protein levels were quantified (right). (E) HOS/143B cells received 24 h of 4‐IPP treatment, which was followed by qRT‐PCR‐based detection of the mRNA level of CDK9. (F) HOS/143B cells were transfected with the MIF overexpression plasmid and subsequently treated with 40 μM 4‐IPP for 48 h. Cells were lysed, and proteins were detected by western blotting (left). c‐Myb protein levels were quantified (right). (G) HOS/143B cells were transfected with the MIF knockout plasmid and subsequently treated with 40 μM 4‐IPP for 48 h. Cells were lysed, and proteins were detected by western blotting (left). c‐Myb protein levels were quantified (right). (H) HOS/143B cells were treated with different concentrations of 4‐IPP for 24 h, which was followed by cell lysis; then, the proteins were detected by western blotting (left), and indicated protein levels were quantified (right). (I) HOS and 143B cells were treated with 40 μM 4‐IPP for 24 h, and p65 and CDK9 colocalization was detected by immunofluorescence colocalization. Scale bar, 10 μm. (J and K) ChIP assays were performed with HOS/143B cells stimulated with 40 μM 4‐IPP, and the enrichment of p65 binding to the promoter region of the CDK9 gene was determined by PCR (J) and qRT‐PCR (K). (L) The inhibitory effect of 4‐IPP on p65 binding to the CDK9 promoter region reporter was demonstrated by a dual luciferase assay. (Data were obtained from triplicate experiments and are expressed as the mean ± SD; *p < 0.05, **p < 0.01, ***p < 0.001 for a comparison with the control group or as indicated)

Abnormal activation of the NF‐κB pathway promotes osteosarcoma growth and metastasis.^21^, ^22^ 4‐IPP effectively inhibited NF‐κB pathway activation and p65 phosphorylation and nuclear entry (Figure 4A‐D). Previous literatures have shown that activation of the NF‐κB pathway involves the regulation of multiple proto‐oncogenes.^23^, ^24^, ^25^, ^26^, ^27^ We hypothesized that 4‐IPP‐induced NF‐κB pathway inhibition may involve these proto‐oncogenes. Therefore, we evaluated the changes in several proto‐oncogenes under 4‐IPP treatment, including *c‐Myb*, *KRAS*, *c‐Fos*, *HRAS*, *c‐Myc*, *NRAS* and *c‐Jun*. The results showed that *KRAS*, *c‐Fos*, *HRAS*, *c‐Myc*, *NRAS* and *c‐Jun* presented a significant downward adjustment, with *c‐Myb* showing the most significant down‐regulation in HOS cells (Figure 5A). After the HOS/143B cells were treated with 4‐IPP, *c‐Myb* mRNA level showed a significant decrease (Figure 5B). Previous studies^23^, ^28^, ^29^ reported that transcriptional regulation of c‐Myb was related to the CDK9 and NF‐κB pathways. We hypothesized that 4‐IPP regulates c‐Myb transcription through the NF‐κB pathway and CDK9. A plasmid of the pCMV6‐entry vector that can overexpress CDK9 was constructed and showed considerable overexpression efficiency in HOS/143B cells (Figure S7A). The overexpression of CDK9 increased the mRNA level of c‐Myb and rescued the down‐regulation of c‐Myb induced by 4‐IPP and Bay 11–7085 in HOS/143B cells (Figure S7B). Then, HOS cells were treated with 4‐IPP, Bay 11–7085 (an inhibitor of NF‐κB activation and phosphorylation of IκBα) and Bay‐1143572 (CDK9 inhibitor) alone or in combination, and c‐Myb showed a significant reduction (Figure S7C). In vitro recruitment of p65 and CDK9 to the c‐Myb promoter was further investigated by ChIP in HOS/143B cells. CDK9 and p65 bound to the predicted promoter of c‐Myb under basal conditions but not under 4‐IPP treatment in HOS/143B cells (Figure 5C). Overexpression of CDK9 and p65 could obviously rescue the inhibition effect of 4‐IPP in c‐Myb protein level (Figure 5D). And the expression level of CDK9 mRNA was inhibited by the 4‐IPP treatment in a concentration‐dependent manner (Figure 5E). The exogenous up‐regulation of MIF expression was found to reverse the 4‐IPP treatment‐induced decreased in c‐Myb proteins (Figure 5F). And the knockout of MIF expression was found to abolish the 4‐IPP treatment‐induced decreased in the expression of c‐Myb proteins (Figure 5G). A plasmid of the pCMV6‐entry vector that can overexpress c‐Myb was constructed and stably transfected into 143B and HOS cells (Figure S7D) for CCK‐8 (Figure S7E) and colony formation (Figure S7F) assays. Exogenous up‐regulation of c‐Myb expression was found to block the 4‐IPP treatment‐induced impairment of proliferation ability. Knockout of CDK9 and p65 genes by CRISPR/Cas9 plasmid can also reduce the protein level of c‐Myb (Figure S7G,H). The above results indicate that 4‐IPP can regulate the transcriptional expression of c‐Myb through NF‐κB/CDK9.

We further studied how NF‐κB and CDK9 interact to regulate c‐Myb transcriptional expression. First, the expression level of CDK9 mRNA was inhibited by the 4‐IPP treatment (Figure 5E). At the protein level, 4‐IPP inhibited the accumulation of CDK9 and the phosphorylation of Rpb1 CTD (Ser2) and Rpb1 CTD (Ser5) (Figure 5H). The combined application of 4‐IPP and Bay 11–7085 also inhibited CDK9 mRNA and protein levels (Figure S7I,J). The combination of p65 and CDK9 was further investigated by immunofluorescence colocalization in HOS/143B cells. As shown in Figure 5I, 4‐IPP treatment obviously reduced the level of CDK9 expression and the nuclear translocation of p65, leading to a colocalized reduction in CDK9 and p65 in the nucleus. The above results confirm the role of the transcription factor p65 in regulating the transcriptional expression of CDK9. In vitro recruitment of p65 to the CDK9 promoter was further investigated by ChIP in HOS/143B cells (Figure S8A). As shown by the results of ChIP analysis, 4‐IPP stimulation reduced the physical binding of p65 to the promoter region of the CDK9 gene (Figure 5J,K). According to the luciferase reporter assay, the transcriptional regulation of CDK9 by p65 was significantly inhibited by 4‐IPP, which was reversed when the CDK9 promoter was mutated (Figure 5L). Further studies were conducted to clarify whether the overexpression of CDK9 could rescue the anti‐tumour effect of 4‐IPP on osteosarcoma. According to the CCK‐8 (Figure S8B) and colony formation (Figure S8C) assay, the exogenous up‐regulation of CDK9 expression blocked the 4‐IPP treatment‐induced impairment of proliferation ability. Then, according to wound‐healing assays (Figure S8D) and Transwell migration and Matrigel invasion assays (Figure S8E), the exogenous up‐regulation of CDK9 expression reversed these impairments. We also tested the correlation between MIF and CDK9/CTD. After the overexpression of MIF in HOS/143B cells, the expression of CDK9 and phosphorylation of the Rpb1 CTD were significantly increased. The exogenous up‐regulation of MIF expression was found to reverse the 4‐IPP treatment‐induced inhibition of CDK9 and phosphorylation of Rpb1 CTD (Figure S8F). After MIF knockout in HOS/143B cells, CDK9 and phosphorylation of the Rpb1 CTD were significantly decreased, and 4‐IPP also lost its effect on CDK9 and phosphorylation of Rpb1 CTD (Figure S8G). Hence, it is concluded that 4‐IPP hinders osteosarcoma cell development by blocking the formation of the p65/CDK9 transcriptional complex, thereby reducing the transcription of its direct target gene c‐Myb.

### 3.5 STUB1 is identified as the E3 ligase of MIF and mediates 4‐IPP‐induced MIF protein ubiquitylation and proteasomal degradation

**FIGURE 6:**
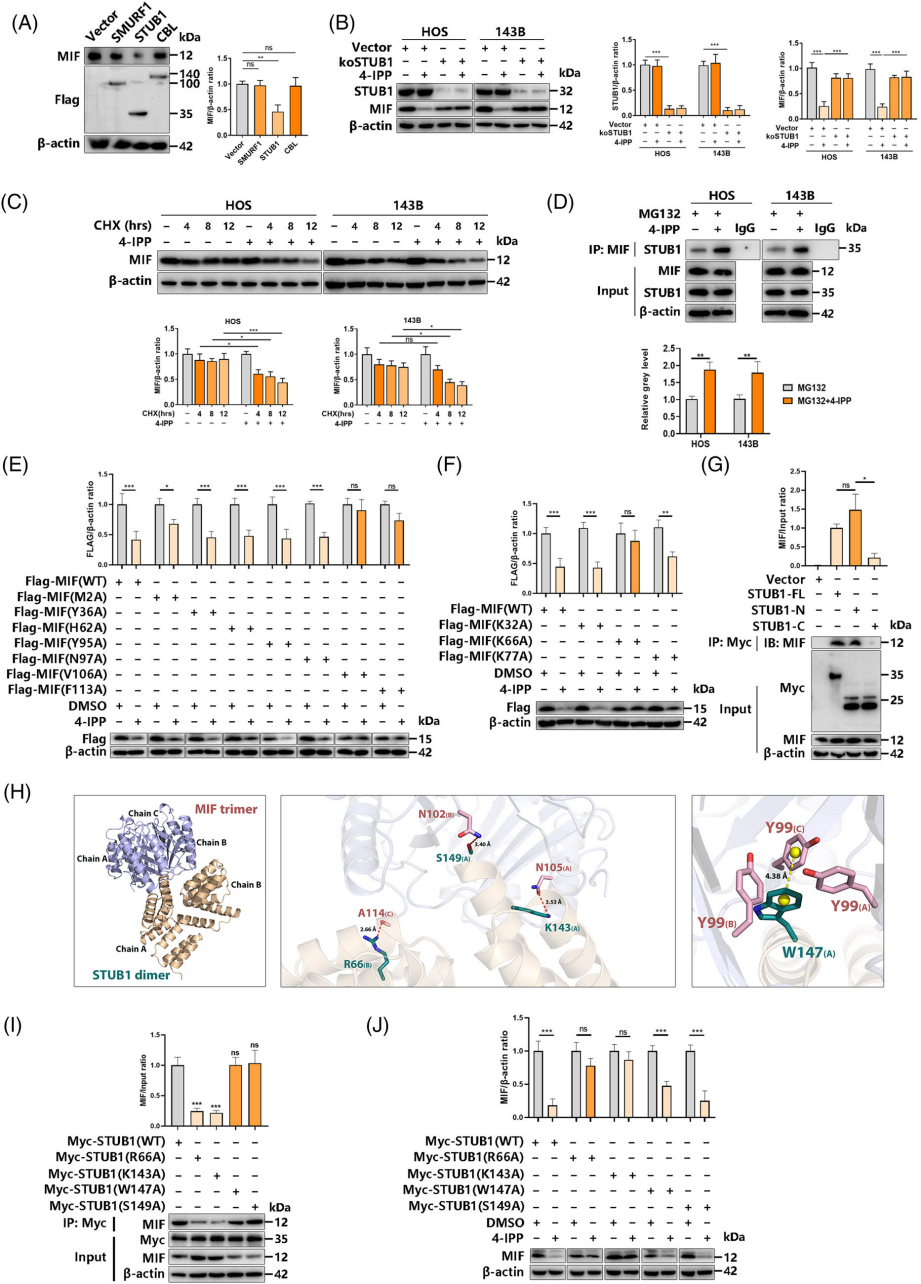
The migration inhibitory factor (MIF) inhibitor 4‐IPP induces MIF degradation via the E3 ubiquitin‐protein ligase STUB1. (A) 293T cells were transfected with the indicated plasmids for 48 h, which was followed by cell lysis; then, proteins were detected by western blotting (left), and MIF protein levels were quantified (right). (B) HOS and 143B cells were transfected with the STUB1 knockout plasmid and subsequently treated with 4‐IPP for 48 h. Cells were lysed, and proteins were detected by western blotting (left). MIF protein levels were quantified (right). (C) HOS and 143B cells were incubated for 1 h with 100 μM cycloheximide followed by 100 μM cycloheximide supplemented with vehicle or 40 μM 4‐IPP for the indicated time. Proteins were detected by western blotting (left), and MIF protein levels were quantified (right). (D) HOS/143B cells were stimulated with 40 μM of 4‐IPP for 12 h, and total cellular proteins extracted were subjected to immunoprecipitation using specific antibodies against MIF. Immunoprecipitates were then subjected to western blot analyses using specific antibodies against STUB1. (E) 293T cells were transfected with the indicated MIF mutation plasmids for 24 h and treated with 4‐IPP for 24 h, which was followed by cell lysis. Then, proteins were detected by western blotting (bottom), and Flag‐MIF protein levels were quantified (top). (F) 293T cells were transfected for 24 h with the indicated MIF mutation plasmids and then treated with 4‐IPP for 24 h, which was followed by cell lysis. Then proteins were detected by western blotting (bottom), and Flag‐MIF protein levels were quantified (top). (G) 293T cells were transfected for 24 h with the indicated STUB1 protein truncation plasmids, followed by cell lysis. Then, proteins were detected by coimmunoprecipitation and western blotting (bottom), and MIF protein levels were quantified (top). (H) Binding mode of the STUB1 dimer (positions 23–152) on the MIF trimer (positions 1–118) predicted by docking. Overall structure of the STUB1 dimer bound to the MIF trimer in cartoon view. The chain identifiers of each protein are labelled (left); STUB1 and MIF are marked in wheat and light blue, respectively. Detailed interaction network between the STUB1 dimer and MIF trimer. Sticks denote the key residues of MIF (pink) and STUB1 (deep teal), with the chain identifiers of residues shown. Red dashed lines denote H‐bonds, with their distances (acceptor to donor heavy atom) labelled (middle). W147 of STUB1 forms edge‐to‐face π stacking with Y99 of MIF. The centroids of aromatic rings are represented as yellow balls, and the distance between the centroids is labelled (right). (I) 293T cells were transfected for 24 h with the indicated STUB1 mutation plasmids, followed by cell lysis. Then, proteins were detected by coimmunoprecipitation and western blotting (bottom), and MIF protein levels were quantified (top). (J) 293T cells were transfected for 24 h with the indicated STUB1 mutation plasmids, followed by 24 h of 4‐IPP treatment and cell lysis. Then, proteins were detected by western blotting (bottom), and Flag‐MIF protein levels were quantified (top). (Data were obtained from triplicate experiments and are expressed as the mean ± SD; *p < 0.05, **p < 0.01, ***p < 0.001 for a comparison with the control group or as indicated)

Given the inconsistency of MIF mRNA and protein expression (Figure 1I‐K), we further verified that blocking proteasome‐mediated protein degradation can rescue the 4‐IPP‐induced reduction of MIF protein (Figure 1L). To determine the E3 ligase of MIF, the E3 substrate network of the entire proteome was predicted by the integrated bioinformatics platform UbiBrowser^30^ with support from the naive Bayesian network (http://ubibrowser.ncpsb.org). We screened and listed the five E3 ligases with the highest confidence score among 31 predicted E3 ligases when MIF was the substrate (Figure S9A,B). The first three E3s (SMUPF1, STUB1 and CBL) were overexpressed, and the MIF level was determined to further confirm the E3 ligase of MIF. Only STUB1 overexpression led to a significant decrease in MIF protein levels (Figure 6A). Furthermore, in STUB1 knockdown cells, 4‐IPP caused no reduction in the MIF protein level (Figure 6B), thus revealing its role in driving 4‐IPP MIF proteasomal degradation through STUB1‐mediated ubiquitination. Next, we used cycloheximide (CHX) to inhibit protein synthesis in HOS/143B cells and tested the effect of 4‐IPP on the MIF level. Consistently, the combined effect of CHX and 4‐IPP led to lower protein expression, higher ubiquitination and a shorter half‐life of MIF (Figure 6C). The results of co‐immunoprecipitation experiments show that 4‐IPP could promote the binding of MIF and p65 or CD74 (Figure 6D). These results suggest that 4‐IPP was associated with STUB1‐mediated MIF proteasomal degradation.

Given the findings illustrated above, 4‐IPP was identified as a possible inverse MIF agonist that weakens MIF stability. A previous study^31^ identified seven major interaction partners for bound 4‐IPP, including MET A2, TYR A36, HIS A62, VAL A106, PHE A113, TYR B95, and ASN B97. Here, we mutated these sites to explore the exact site of 4‐IPP‐mediated proteasomal degradation, and the V106 and F113 sites were identified as key sites for 4‐IPP‐mediated MIF degradation (Figure 6E). We then explored the ubiquitination site of MIF with only three lysine sites for K32, K66 and K77. As shown in Figure 6F, the mutation of K66 obviously rescued the 4‐IPP‐induced MIF protein reduction. Then, we studied the interaction between MIF and STUB1. According to the structural distribution of STUB1, including the tetratricopeptide repeat domain and U‐box domain, we constructed different regions of STUB1 to study the scope of action of STUB1 with MIF (Figure S9C). The N‐terminus of STUB1 showed a stronger ability to combine with MIF than the C‐terminus (Figure 6G). To verify the interaction between MIF and STUB1, we first assessed the binding site of MIF and STUB1 using a virtual docking assay. Four potential key binding sites were found: R66, K143, W147 and S149 (Figure 6H). Furthermore, we mutated these sites to evaluate the change in the interaction of MIF and STUB1. As shown in Figure 6I, the mutation of R66 and K143 obviously blocked the binding of MIF and STUB1 as well as the degradation of MIF protein. Thus, the results of the 4‐IPP treatment indicated that the mutation of R66 and K143 could reverse 4‐IPP‐induced MIF protein degradation (Figure 6J). In summary, the interaction and direct binding of MIF with its E3 ligase STUB1 are promoted by 4‐IPP.

To clarify whether STUB1 plays an important role in the 4‐IPP‐induced anti‐tumour effect, stable transfection of HOS and 143B cells was achieved with the STUB1 CRISPR‐Cas9 knockout plasmid of the LentiCRISPR vector. CCK‐8 (Figure S9D) and colony formation (Figure S9E) assay were performed to evaluate the proliferation ability of osteosarcoma cells. Knockout of STUB1 blocked the 4‐IPP‐induced impairment of proliferation ability. Then, through wound healing assays (Figure S9F) and Transwell migration/invasion assays (Figure S9G), we evaluated the metastatic ability of osteosarcoma. Knockout of STUB1 was found to reverse the 4‐IPP‐induced impairment of migration and invasion abilities. These findings suggest that STUB1 knockout could abolish the anti‐tumour effects of 4‐IPP treatment.

### 3.6 MIF degradation by 4‐IPP suppresses osteosarcoma tumourigenesis and metastasis in vivo

**FIGURE 7:**
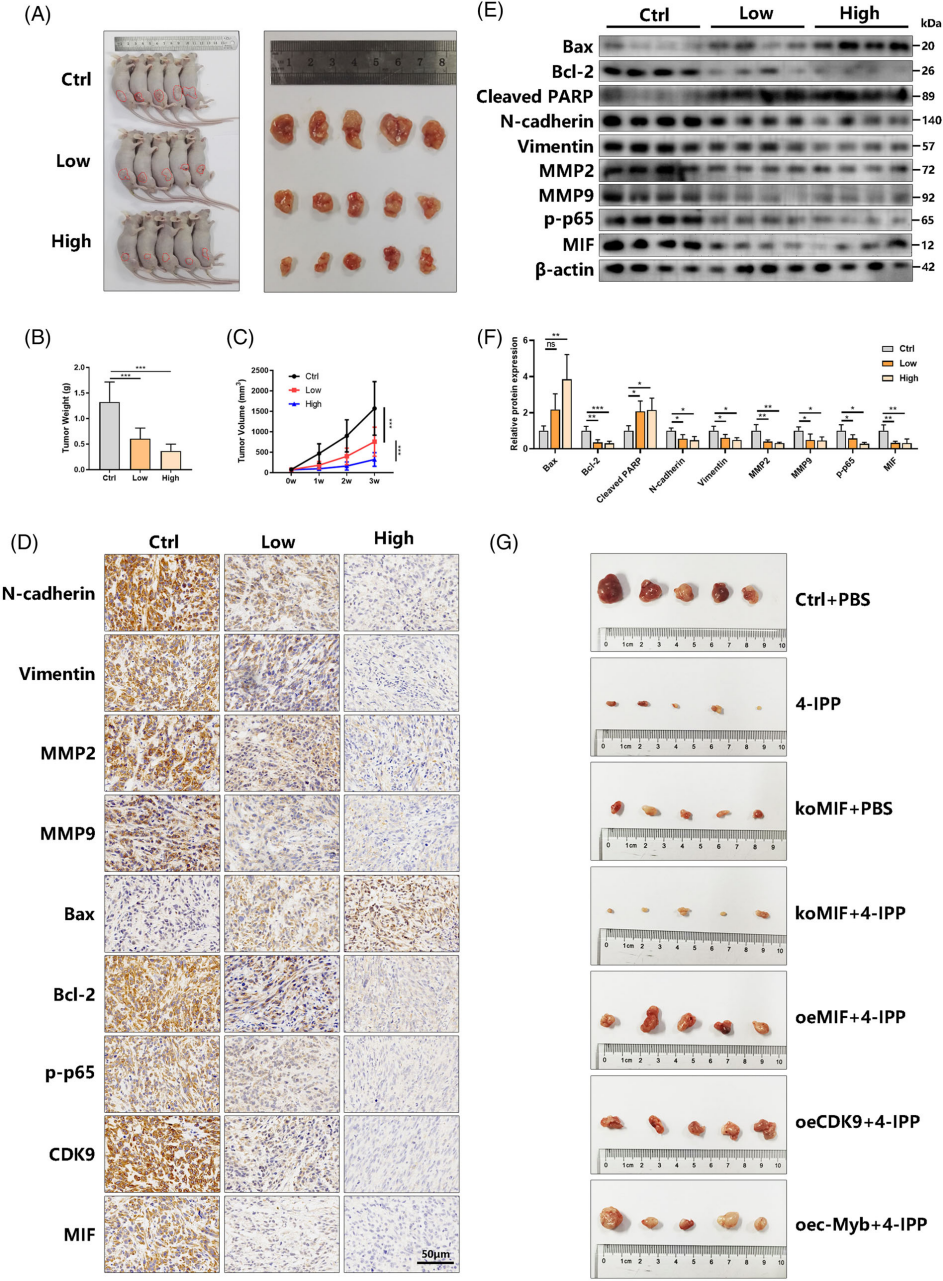
Migration inhibitory factor (MIF) degradation by 4‐iodo‐6‐phenylpyrimidine (4‐IPP) suppresses osteosarcoma tumourigenesis and metastasis in vivo. (A) Nude mice were used to establish an HOS‐derived xenograft model. After they were sacrificed (*n* = 5), xenograft tumours were separated from the surrounding tissue. (B) The tumour volume was measured every 7 days. (C) Finally, we determined the average tumour weight in each group. (D) Representative pictures of the indicated proteins detected by immunohistochemistry. Scale bar, 50 μm. (E) The expression levels of the indicated proteins in tumours from different groups (*n* = 4) were detected by western blotting. (F) Quantification of the indicated protein levels. (G) HOS cells were stable transfected with indicated plasmids. Nude mice were used to establish an HOS‐derived xenograft model, and tumours were separated from the surrounding tissue after the mice were sacrificed (*n* = 5). (Data are expressed as the mean ± SD; *p < 0.05, **p < 0.01, ***p < 0.001 compared with the control group or as indicated)

An HOS‐derived xenograft model in nude mice was built to determine whether 4‐IPP could reduce osteosarcoma growth in vivo. 4‐IPP (0, 5 or 20 mg/kg for Ctrl group, low group and high group, respectively) was intraperitoneally administered every other day for 3 weeks without toxicity (Figure S10A,B), and it obviously decreased the tumour size and weight compared to those in the control group (Figure 7A‐C). Immunohistochemical analysis of the tumour tissues showed changes in the protein expression of MMP9, vimentin, Bax, Bcl‐2, p‐p65, CDK9, MMP2, N‐cadherin and MIF (Figure 7D). The results indicated that N‐cadherin, vimentin, MMP2, MMP9, Bcl‐2, p‐p65, CDK9 and MIF expression was strongly reduced in the 4‐IPP‐treated group while E‐cadherin and Bax expression was increased. Western blotting demonstrated the changes in protein expression of Bax, Bcl‐2, cleaved PARP, N‐cadherin, vimentin, MMP2, MMP9, p‐p65 and MIF (Figure 7E,F). 4‐IPP treatment predominantly reduced Bcl‐2, N‐cadherin, vimentin, MMP2, MMP9, p‐p65 and MIF protein expression while increasing Bax, cleaved PARP expression. 4‐IPP treatment predominantly reduced MIF content in serum (Figure S10C). Moreover, we used HOS cells to explore the effects of 4‐IPP on the osteosarcoma lung metastasis model in vivo. In Figure S10D suggests that 4‐IPP treatment obviously reduced the lung metastatic tumour number. In order to further verify the specific role of MIF/CDK9/c‐Myb axis in 4‐IPP inhibition effect in osteosarcoma, HOS cells were stably transfected with MIF knockout/MIF overexpression/CDK9 overexpression/c‐Myb overexpression plasmids to establish xenograft model in nude mice, and followed by PBS or 20 mg/kg 4‐IPP treatment. As shown in Figure 7G and Figure S10E,F, compared with the 4‐IPP treatment group, there is no difference in the inhibitory effect of 4‐IPP on osteosarcoma after MIF knockout. The overexpression of MIF/CDK9/c‐Myb can significantly reverse the therapeutic effect of 4‐IPP on osteosarcoma. These data confirm that 4‐IPP induces the degradation of MIF protein to diminish the metastasis of osteosarcoma. These findings indicate that 4‐IPP has potential value in clinical trials for patients suffering from lung metastases.

### 3.7 Extracellular MIF release inhibition by 4‐IPP suppressed the osteosarcoma‐induced osteolysis

**FIGURE 8:**
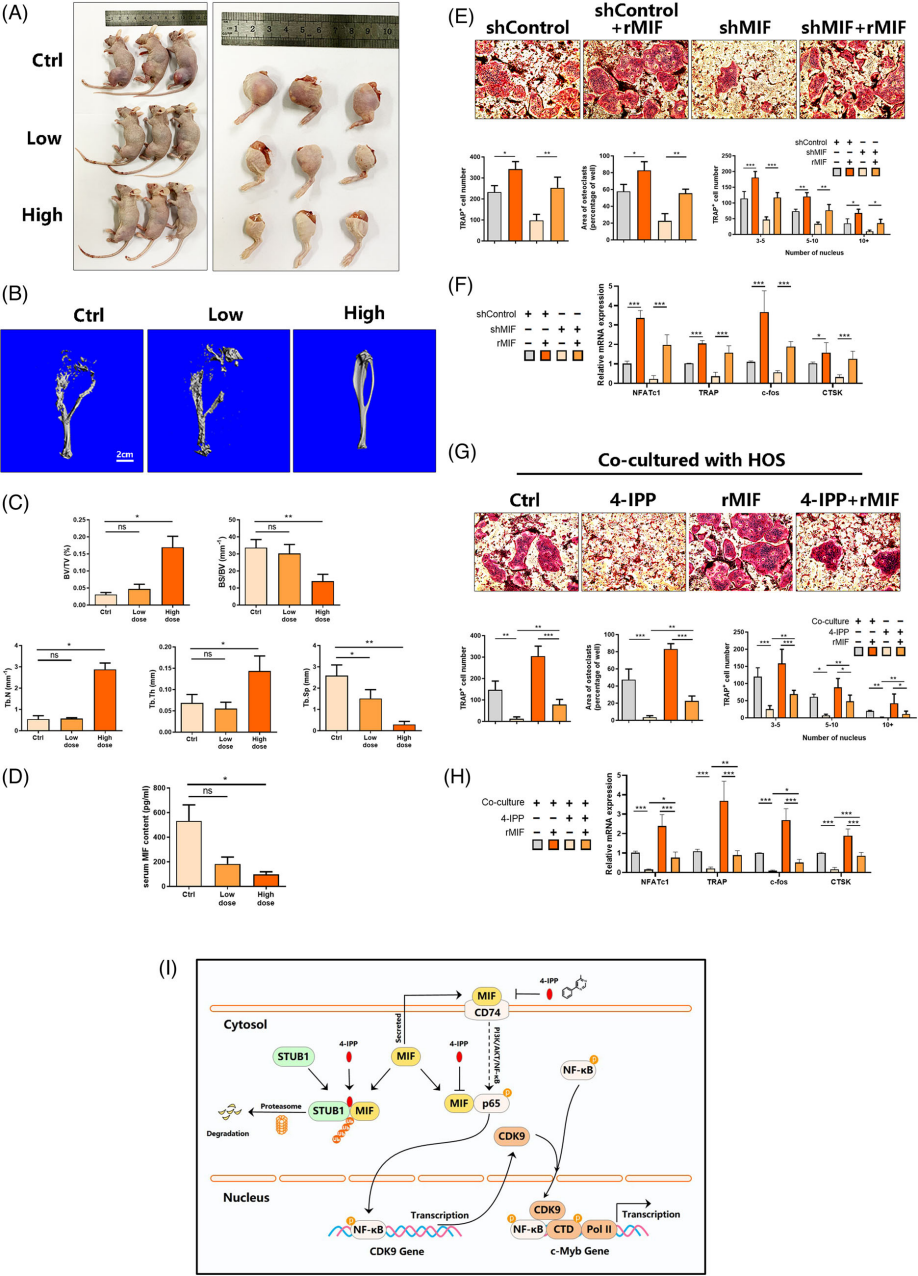
Extracellular migration inhibitory factor (MIF) release inhibition by 4‐iodo‐6‐phenylpyrimidine (4‐IPP) suppressed osteosarcoma‐induced osteolysis. (A) An HOS‐derived orthotopic model was established in nude mice, and tumours were separated from the surrounding tissue after the mice were sacrificed (*n* = 3). (B) Representative 3D μCT reconstructions of the tibial bone of nude mice from different groups. (C) Bone microstructural parameters, including the trabecular spacing (Tb.S.), trabecular thickness (Tb.Th.), trabecular number (Tb.N.), bone volume relative to total volume (BV/TV), and bone surface relative to bone volume (BS/BV). (D) Serum MIF content in different groups. (E) Conditioned medium (CM) was obtained from the supernatant of HOS cells transfected with the vector plasmid or MIF shRNA plasmid. RANKL (50 ng/ml) was used to stimulate BMMs, which were cultured in CM with or without recombinant MIF protein (100 ng/ml) for 5 days, after which multinucleated osteoclasts were fixed and stained for TRAP activity (top). We then obtained the size and number (average area) of TRAP‐positive multinucleated osteoclasts with more than five nuclei and determined the distribution of TRAP‐positive osteoclasts with 3–5, 5–10, or 10+ nuclei (bottom). (F) qRT‐PCR‐based analysis of the gene expression of NFATc1, TRAP, c‐Fos, and CTSK was performed based on the mRNAs extracted from BMM‐derived osteoclasts that were stimulated with RANKL and the indicated CM with or without recombinant MIF protein (100 ng/ml) for 5 days. (G) BMMs were co‐cultured with HOS cells, followed by 5 days of stimulation with 50 ng/ml RANKL and with or without 40 μm 4‐IPP. Multinucleated osteoclasts were subsequently fixed and stained for TRAP activity (top). We then obtained the size and number (average area) of TRAP‐positive multinucleated osteoclasts with over 5 nuclei and determined the distribution of TRAP‐positive osteoclasts with 3–5, 5–10, or 10+ nuclei (bottom). (H) qRT‐PCR‐based analysis of the gene expression of NFATc1, TRAP, c‐Fos, and CTSK was performed based on the mRNAs from BMM‐derived osteoclasts co‐cultured with HOS cells with or without 40 μm 4‐IPP for 5 days. (I) Proposed model of 4‐IPP in the regulation of MIF degradation and osteosarcoma progression and metastasis. 4‐IPP promotes the degradation of the MIF protein via STUB1 E3 ligase. 4‐IPP inhibits the formation of the NF‐κB/CDK9 complex by inhibiting the MIF/NF‐κB pathway, thereby inhibiting the transcription of the proto‐oncogene c‐Myb

Our previous research^11^ revealed that 4‐IPP has a potent inhibitory effect on osteoclast differentiation. Based on the anti‐tumour effect of 4‐IPP in this osteosarcoma study, we further studied whether 4‐IPP can simultaneously exert anti‐tumour and anti‐osteoclast effects in an orthotopic tumour model. 4‐IPP (0, 5 or 20 mg/kg) was intraperitoneally administered every other day for 3 weeks, and it obviously decreased the tumour size compared with that of the control tumours (Figure 8A). The control group nude mice treated with PBS (vehicle) presented high trabecular bone microarchitecture deterioration and significantly less bone mass; thus, an increase in BS/BV and Tb.S and a reduction in BV/TV, Tb.N, and Tb.Th occurred (Figure 8B,C), as shown by tibial 3D reconstruction and morphometric analysis. In contrast, under high‐dose 4‐IPP treatment, the sharp decrease in bone mass and deterioration of the trabecular microarchitecture caused by osteosarcoma were hindered. However, the low‐dose group did not have an obviously significant inhibitory effect. In addition, the MIF content in the serum was significantly suppressed by treatment with 4‐IPP (Figure 8D). We further explored whether MIF derived from osteosarcoma cells has an effect on osteoclast differentiation in vitro. MIF shRNA was used to knock down the MIF gene in HOS cells, and then the condensed supernatant was collected as CM for osteoclast differentiation (Figure S11A). BMMs were cultured with CM from HOS cells transfected with control shRNA or MIF shRNA, and then recombinant MIF protein was added. As shown in Figure 8E, compared to BMMs cultured with control CM, BMMs cultured with MIF knockdown CM had a markedly weakened capacity to differentiate into TRAP‐positive multinucleated osteoclasts; however, recombinant MIF protein obviously rescued the impaired osteoclast differentiation ability. Consistently, the qPCR results showed that the gene expression of c‐Fos, TRAP, NFATc1 and CTSK was significantly reduced in BMMs cultured with MIF knockdown CM but rescued by rMIF protein (Figure 8F). Then, we co‐cultured BMMs and HOS cells with or without 4‐IPP and rMIF. The results showed that 4‐IPP could still significantly inhibit osteoclast differentiation, while rMIF could promote osteoclast differentiation and partially rescue the inhibitory effect of 4‐IPP under co‐culture conditions (Figure 8G). Consistently, the qPCR results showed that the gene expression of c‐Fos, TRAP, NFATc1 and CTSK was significantly reduced by 4‐IPP but partly rescued by the rMIF protein (Figure 8H). The above results indicate that 4‐IPP can impair the promoting effect of osteosarcoma‐derived MIF on osteoclast differentiation in vivo and in vitro.

## 4 DISCUSSION

In this study, we provide evidence that the MIF inhibitor 4‐IPP has an anti‐tumour effect on osteosarcoma by promoting the degradation of the MIF protein and inhibiting the transcription of c‐Myb through hampering the formation of the downstream NF‐κB/CDK9 complex in osteosarcoma (Figure 8I). First, 4‐IPP promotes MIF degradation via the E3 ubiquitin‐protein ligase STUB1; moreover, 4‐IPP inhibits the formation of the NF‐κB/CDK9 complex through its inhibitory effect on the NF‐κB pathway, thereby inhibiting the transcription of the proto‐oncogene c‐Myb.

In osteosarcoma, MIF activates the RAS/MAPK pathway to promote osteosarcoma cell proliferation and migration.^8^ Our previous study^11^ verified that MIF promotes osteoclast differentiation by activating the NF‐κB pathway during osteoclast differentiation and identified the MIF inhibitor 4‐IPP as a potent osteoclast inhibitor. Based on the above research, we wondered whether MIF inhibitors can simultaneously exert anti‐tumour and anti‐osteoclastogenic effects. For this reason, we intended to clarify how 4‐IPP affects osteosarcoma. In the pre‐experimental phase, we tested several inhibitors, including ISO‐1, 4‐IPP, CPSI‐1306, and Chicago sky blue 6B, and 4‐IPP was identified as the most effective inhibitor (data not shown). In subsequent experiments, we found that high concentrations of 4‐IPP could down‐regulate the expression of MIF mRNA, while low concentrations of 4‐IPP could up‐regulate the expression of MIF mRNA. At the protein level, all concentrations of 4‐IPP significantly reduced the level of MIF protein expression. Based on existing research related to small molecule‐induced protein degradation,^32^, ^33^ we consider that 4‐IPP may also act as a molecular glue, involved in the degradation of the MIF protein. We identified STBU1 as a key E3 ligase in the proteasomal degradation of MIF, and it is related to 4‐IPP‐induced MIF protein degradation. Schulz et al.^34^ reported that the ensuing proteasome‐dependent MIF degradation is subject to the mediating effect of the STUB1, which is consistent with our result. We also identified the V106 and F113 sites as the key sites for 4‐IPP mediating MIF degradation. The K66 site was the ubiquitination site of MIF in STUB1‐mediated MIF degradation. To study the interaction between MIF and STUB1, we assessed the binding site of MIF and STUB1 through a virtual docking assay. We performed a docking study of the binding mode between the MIF trimer and STUB1 dimer based on the HDOCK server.^35^, ^36^, ^37^, ^38^, ^39^ According to the docking score provided by the HDOCK server, the best predicted binding mode with the lowest docking score was selected for analysis. The best predicted binding mode was visualized, analysed and mapped using the PyMOL program (http://www.pymol.org). The docking results were analysed by the PyMOL program, through which the interacting residues were determined. Thus, all the interacting residues were identified directly from docking. Docking is usually used to predict the binding modes of small molecules binding to proteins, in which only the small molecules are considered flexible. Because proteins are usually considered rigid in the docking process, many docking algorithms are not suitable for predicting binding complexes between proteins. However, the HDOCK server is a protein‐protein and protein‐DNA/RNA docking server based on a hybrid algorithm of template‐based modelling and ab initio free docking and performs very well in protein‐protein docking.^37^ Then, the R66 and K143 sites of STUB1 were identified as the key sites for the interaction between MIF and STUB1. The Y99 site of MIF also interacts with STUB1, but its mutation has no effect on the degradation of MIF. However, other studies have reported the effect of Y99 on the catalytic activity of MIF and MIF‐induced activation of CD74.^40^, ^41^

MIF is reported to be related to multiple signaling pathways, including MAPK,^8^ NF‐κB^20^ and PI3K/AKT.^18^ In this study, we tested all of the above signaling pathways and identified PI3K/AKT/NF‐κB as a direct downstream pathway of MIF that is inhibited by 4‐IPP. At the same time, 4‐IPP also inhibits MAPK pathways, including JNK, ERK and p38. After the NF‐κB pathway was identified as a direct downstream target of MIF by 4‐IPP, we further investigated whether a certain exact proto‐oncogene is regulated by NF‐κB in osteosarcoma. Previous studies^23^, ^28^, ^29^ reported that transcriptional regulation of c‐Myb was related to the CDK9 and NF‐κB pathways. When transcribed through its p50 subunit, heterodimeric NF‐κB p50 binds to nascent stem‐loop RNA and interacts with its p65 subunit via the P‐TEFb complex. NF‐κB‐PTEFb forms a multiprotein complex, which is the main driving force of transcription.^42^, ^43^, ^44^ Then, we speculated that the formation of the transcription complex of NF‐κB‐PTEFb controls the transcriptional regulation of c‐Myb. Further experiments found that the CDK9 protein level was significantly decreased by 4‐IPP treatment. The IκBα inhibitor Bay 11–7085 was applied, and the results indicate that transcription of CDK9 is also regulated by the NF‐κB pathway, which was also confirmed by ChIP assay. Then, immunofluorescence colocalization was used to determine the colocalization of p65 and CDK9 in the nucleus. Furthermore, the recruitment of p65 and CDK9 to the c‐Myb promoter was confirmed by ChIP assay. Thus, transcriptional regulation of c‐Myb by the NF‐κB‐PTEFb complex in osteosarcoma has been proven. It is worth noting that the verification of MIF downstream of PI3K/AKT/NF‐κB/P‐TEFb/c‐Myb is based on the regulation of the content of MIF itself by 4‐IPP. Subsequent pathway changes related to MIF are not absolute for the MIF inhibitor 4‐IPP. The upstream and downstream relationship of the pathway is based on previous literature. This does not mean that this is just a single linear relationship, as it may also be a network relationship. MIF itself and MIF inhibitors may also have an impact on the position of nodes in the network relationship. Follow‐up research is needed to verify this possibility.

Emerging evidence has indicated that MIF is an important link between inflammation and malignant progression in the tumour microenvironment.^45^, ^46^ In this study, based on the special environment of osteosarcoma, we focused on the cell–cell communication between osteosarcoma cells and osteoclasts in the final stage of the research. The results demonstrated that 4‐IPP could not only directly inhibit osteosarcoma cells and the differentiation of osteoclasts but also directly interrupt cell communication between the two, thus exerting a dual inhibitory effect. In an in vitro experiment, 4‐IPP effectively inhibited osteosarcoma‐mediated osteoclastogenesis and was rescued by the rMIF protein. In the in vivo experiment, high‐dose 4‐IPP treatment almost completely rescued the osteolysis caused by osteosarcoma, which may be related to this dual inhibitory effect. Although low‐concentration drug therapy has a certain inhibitory effect on tumours, it has a limited ability to mitigate osteolysis, suggesting that tumour suppression has a higher priority. However, in the tumour microenvironment of osteosarcoma, how exactly MIF affects osteoclast production remains to be explored. RANK/RANKL/OPG axis, as a key factor in osteoclast formation, is it regulated by MIF? This is one of the possible potential mechanisms. In addition, the inhibitory concentration of 4‐IPP on osteosarcoma cells in vitro is μM level. This is not an ideal low concentration. However, our in vivo experiments can also achieve the ideal tumour suppression effect with low‐concentration 4‐IPP doses. This also suggests the potential effect of 4‐IPP on angiogenesis or tumour immunity. Although this study included macrophages (bone marrow‐derived macrophages), we did not examine MIF‐mediated tumour inflammation or tumour‐associated macrophages. The role of MIF and its receptors in the tumour microenvironment has been reported in other tumours.^47^, ^48^, ^49^ In malignant gliomas, autocrine MIF counteracted NK and cytotoxic T‐cell‐mediated tumour immune surveillance.^47^ In melanoma, MIF/CD74 cannot only regulate the expression of PD‐L1 but also affect the anti‐tumour immune response of macrophages and dendritic cells.^48^, ^49^ In addition, studies have reported other receptors of MIF, including CD44, CD74, CXCR4, CXCR2 and CXCR7.^9^, ^50^, ^51^, ^52^ Among them, CD74 may also be combined with CD44 or CXCR4 to form a complex receptor.^53^, ^54^ Studies have also reported the potential role of these receptors in osteosarcoma,^55^, ^56^, ^57^, ^58^ and whether these effects are related to MIF remains to be studied. These reports all suggest the possible immune effect of MIF and its receptors in osteosarcoma. Our research initially explores the role of MIF/CD74 in osteosarcoma. 4‐IPP can block the combination of MIF and CD74, thereby inhibiting its downstream pathway NF‐κB. But whether other receptors may play other roles, we still have not resolved this question. The crucial role of the proinflammatory factor MIF in the tumour microenvironment and tumour immunity is hence speculated. Further related experiments are expected to be carried out.

Although many studies have examined the role of MIF in tumours, little is known about DDT (MIF‐2 or DDT), which is a close structural homologue to MIF.^59^, ^60^ Furthermore, an analysis of the signaling properties of the two proteins showed that they work cooperatively and that neutralization of D‐DT in vivo significantly decreases inflammation.^60^ In addition, 4‐IPP is a covalent tautomerase inhibitor of both DDT and MIF.^61^, ^62^ It is possible that the effect of 4‐IPP on MIF is also existed on DDT. Furthermore, the human genome encodes a gene known as DDT like (DDTL). DDTL is located in close proximity to DDT and MIF, and approximately 80% of the gene sequence of DDTL overlaps with that of DDT. To date, nothing is known about the biological function of DDTL or its expression in osteosarcoma. The role of DDT and DDTL in osteosarcoma remains to be explored. In this study, overexpression of MIF can reverse the inhibitory effect of 4‐IPP on osteosarcoma, and knockout of MIF can abolish this effect. And the mRNA expression of MIF is much higher than that of DDT and DDTL. These results could indirectly rule out the potential effects of DDT and DDTL.

## 5 CONCLUSIONS

In summary, our findings show that 4‐IPP is a specific double‐effector drug for osteosarcoma with both anti‐tumour and anti‐osteoclastogenic functions. In addition to its inhibitory effect on the proliferation and metastasis of osteosarcoma cells in vitro, 4‐IPP can also effectively inhibit tumour cell proliferation and osteolysis in vivo. Mechanistically, we demonstrated that the transcriptional regulation of c‐Myb was mediated by MIF through the NF‐κB/P‐TEFb complex in osteosarcoma (Figure 8I). Finally, we found that 4‐IPP can mediate small molecule‐induced MIF protein degradation via the STUB1 E3 ligand, which can lead to advances in the development of therapeutic agents that target malignancies driven by aberrant MIF activity.

## CONFLICT OF INTEREST

The authors declare that they have no conflict of interest.

## Supporting information

Supporting information

Supporting information
