# Supplementary material for: Destabilization of macrophage migration inhibitory factor by 4‐IPP reduces NF‐κB/P‐TEFb complex‐mediated c‐Myb transcription to suppress osteosarcoma tumourigenesis
Source: Clin Transl Med. 2022 Jan 20;12(1):e652. doi: 10.1002/ctm2.652 (PMC8777168; doi:10.1002/ctm2.652)
Supplement: Supplementary file 1 — Supporting information [file CTM2-12-e652-s002.docx]

Supplemental Information

Destabilization of Macrophage Migration Inhibitory Factor by 4-IPP Reduces NF-κB/P-TEFb Complex-Mediated c-Myb Transcription to Suppress Osteosarcoma Tumorigenesis

Lin Zheng, Zhenhua Feng, Siyue Tao, Jiawei Gao, Xiao'an Wei, Bingjie Zheng, Bao Huang, Zeyu Zheng, Xuyang Zhang, Junhui Liu, Zhi Shan, Yilei Chen, Jian Chen, Fengdong Zhao

Supplementary Figure

**
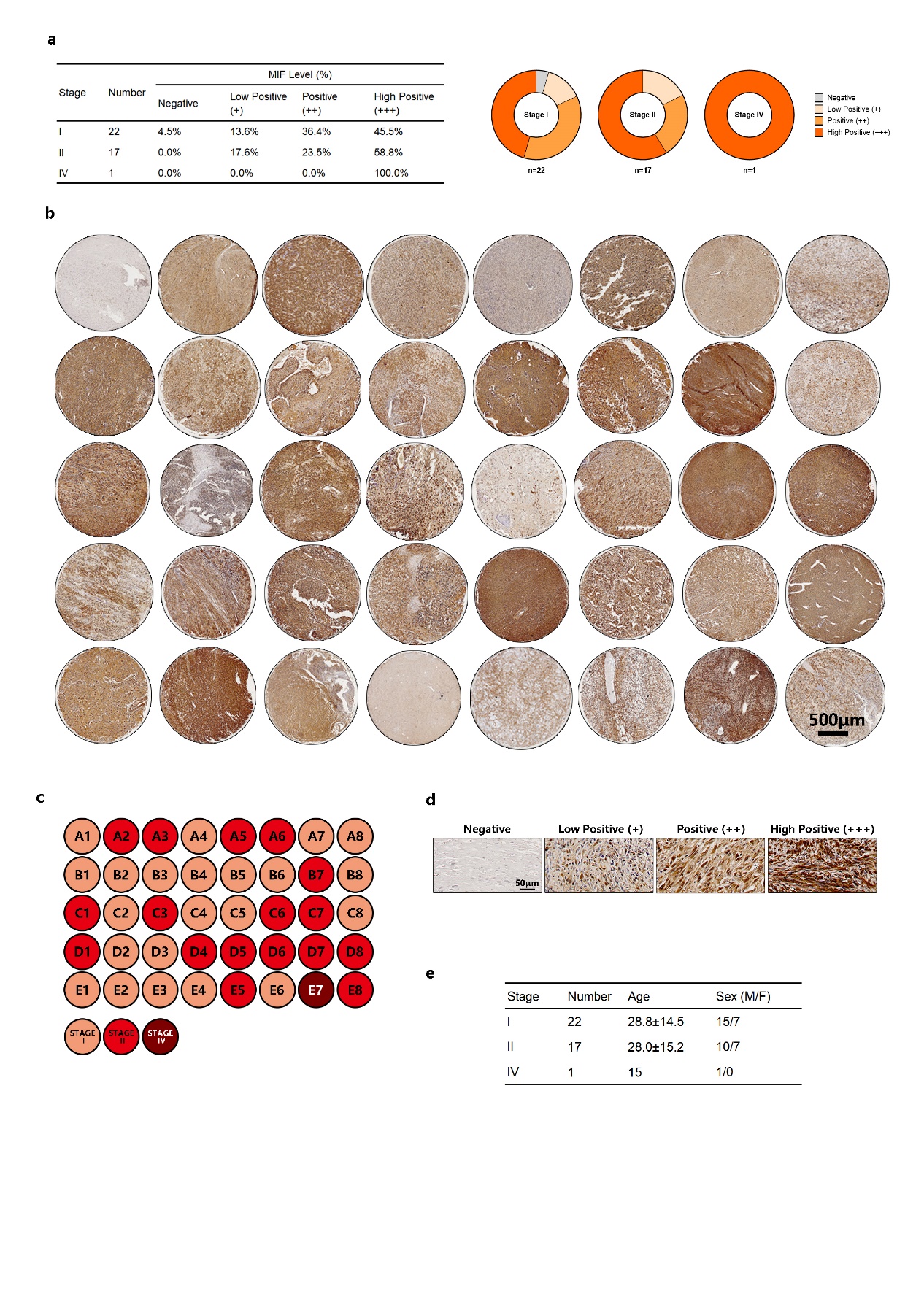
**

**Supplementary Figure 1.** **MIF expression in different stages of osteosarcoma patients.** (a) Immunohistochemical detection of MIF expression was performed in 40 osteosarcoma samples at different stages. (b) Panoramic histochemical images of each individual osteosarcoma sample. (c) Sequence diagram of osteosarcoma samples. (d) Representative images of different ratings of immunohistochemistry. (e) Basic information for patients with osteosarcoma.


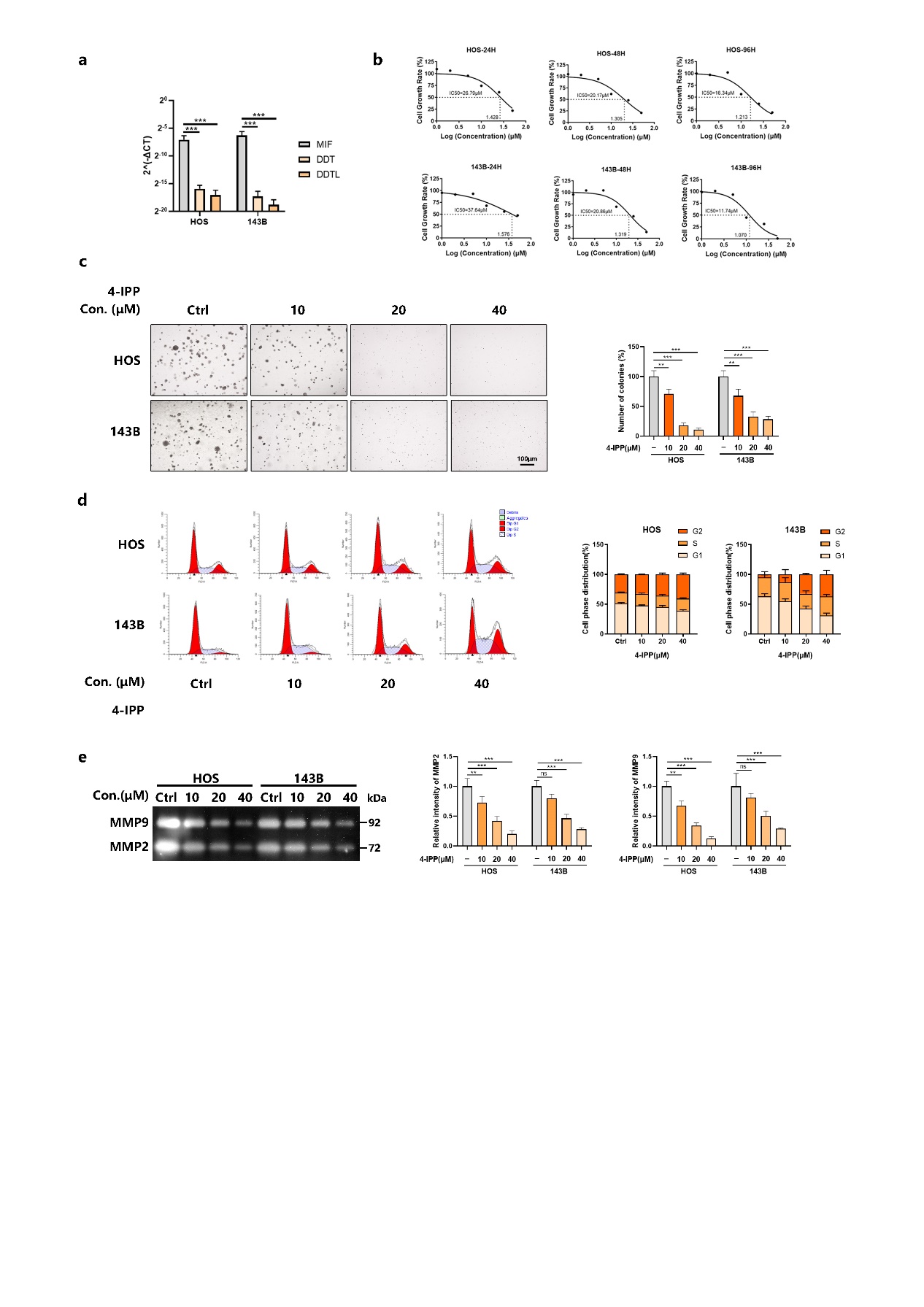


**Supplementary Figure 2.** **4-IPP inhibited the proliferation of HOS and 143B cell lines.** (a) The mRNA expression of *MIF*, *DDT*, and *DDTL* in HOS and 143B was evaluated by qPCR, and the results were analyzed by the 2^(–ΔCT) method, and β-actin was used as the internal reference. (b) Calculated IC50 value of 4-IPP in HOS/143B cells at different times. (c) Proliferation ability of osteosarcoma cells treated with different concentrations of 4-IPP at 14 days is indicated by representative images (left) of the soft agar colony formation assay and quantification of the number of colonies (right). Scale bars, 100 μm. (d) HOS/143B cells were subjected to cell cycle assays, followed by 48 h of 4-IPP treatment (left) and cell cycle distribution (right). (e) HOS/143B cells were treated with different concentrations of 4-IPP, and the supernatant was subjected to detect the activity of MMP2/MMP9 by gelatin zymography assay (left) and quantification was conducted by grayscale analysis. (Data were obtained from triplicate experiments and expressed as the mean ± SD; *p < 0.05, **p < 0.01, ***p < 0.001 compared to the control or as indicated)


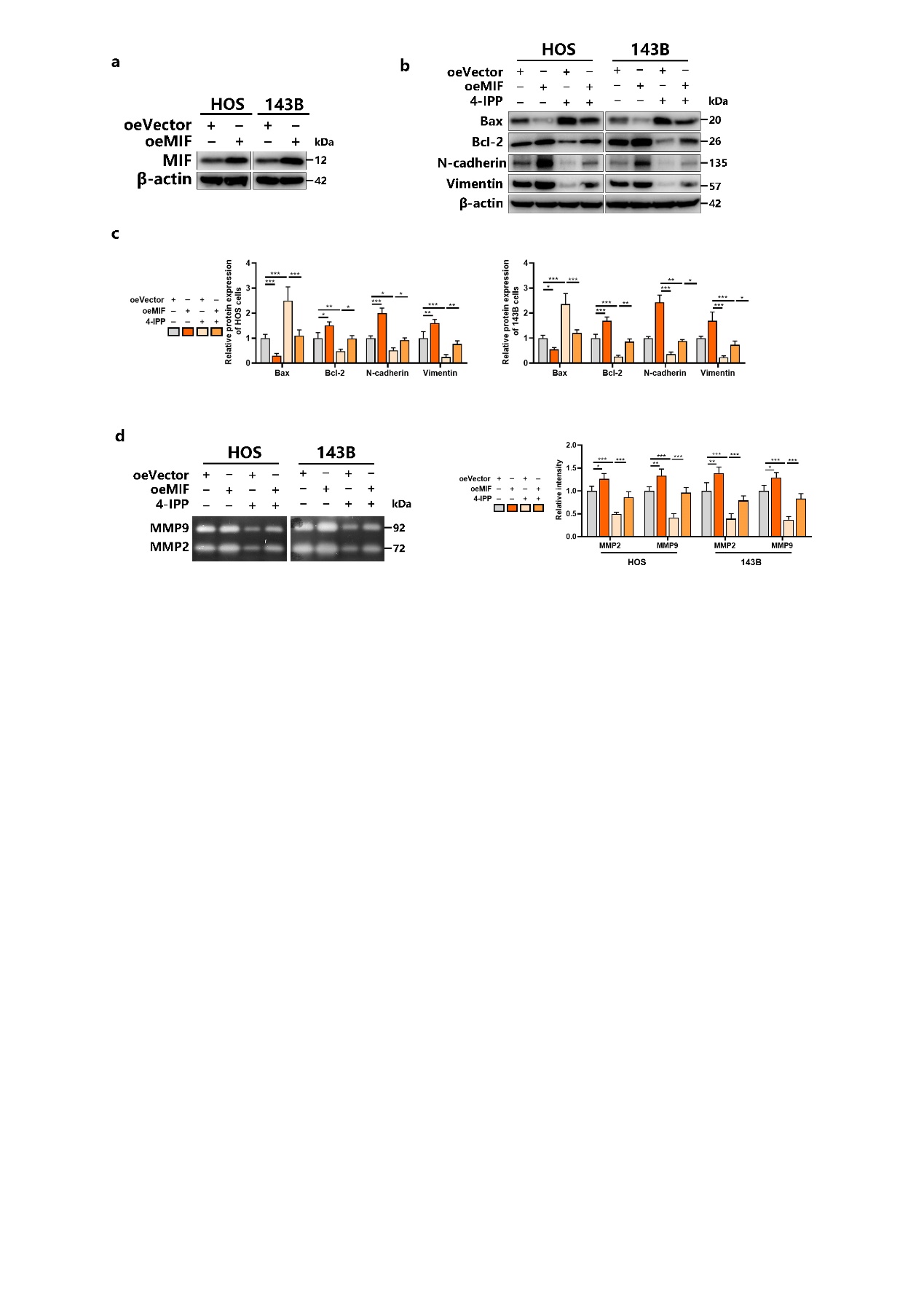


**Supplementary Figure 3.** **MIF overexpression rescues the inhibition of 4-IPP in osteosarcoma.** (a) After stable transfection with the vector plasmid or MIF overexpression plasmid, HOS/143B cells were tested for the transfection efficiency of MIF by western blot. (b) Western blotting was performed to assess the protein expression of Bax, Bcl-2, N-cadherin and Vimentin in HOS/143B cells treated with indicated conditions. (c) Gray levels of indicated protein were quantified and normalized to β-actin using ImageJ. (d) HOS/143B cells were treated with indicated conditions, and the supernatant was subjected to detect the activity of MMP2/MMP9 by gelatin zymography assay (left) and quantification was conducted by grayscale analysis. (Data were obtained from triplicate experiments and expressed as the mean ± SD; *p < 0.05, **p < 0.01, ***p < 0.001 compared to the control or as indicated)


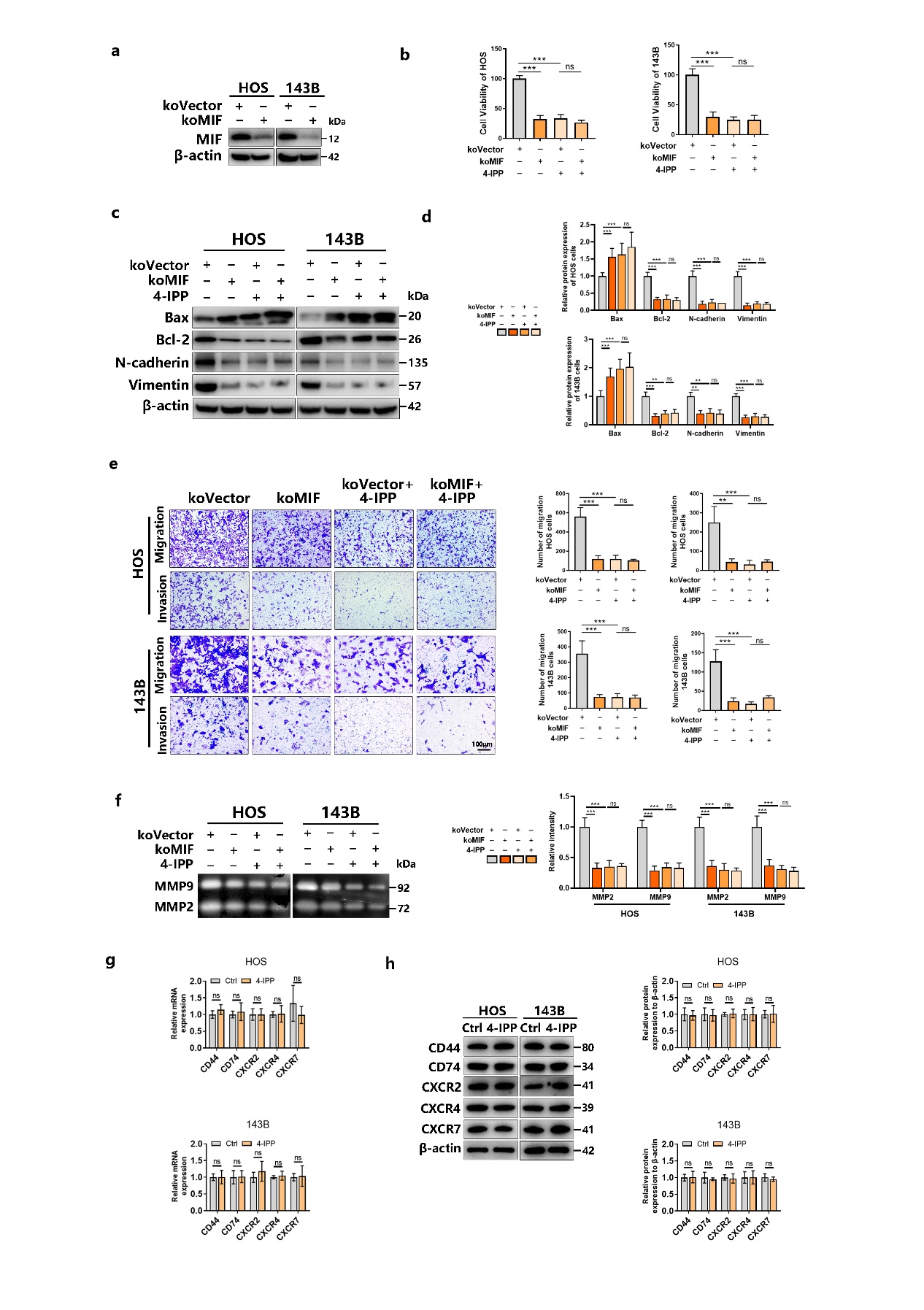


**Supplementary Figure 4. MIF knock down abolished the inhibition of 4-IPP in osteosarcoma.** (a) After stable transfection with the vector plasmid or MIF knockout plasmid, HOS and 143B cells were tested for the knockout efficiency of MIF by western blot. (b) After stable transfection with the vector plasmid or MIF knockout plasmid, HOS and 143B cells were treated with 4-IPP for 48 h, and viability was tested by CCK‐8 assays. (c) Western blotting was performed to assess the protein expression of Bax, Bcl-2, N-cadherin and Vimentin in HOS/143B cells treated with corresponding conditions. (d) ImageJ-based quantification and normalization of the gray levels of the above proteins to that of β‐actin. (e) HOS/143B cells treated with corresponding conditions, and Transwell migration/Matrigel invasion assays were performed to evaluate cell migration and invasion. Scale bars, 100 μm. Number of migrated or invaded cells was quantified. (f) HOS/143B cells treated with corresponding conditions, and the supernatant was subjected to gelatin zymography assay to detect the activity of MMP2/MMP9 (left) and quantification was conducted by grayscale analysis. (g) HOS/143B cells treated with 40μM 4-IPP for 24h, mRNA expression of receptors was detected by qPCR. (h) HOS/143B cells treated with 40μM 4-IPP for 24h, protein expression of receptors was detected by western blot. ImageJ-based quantification and normalization of the gray levels of the above proteins to that of β‐actin. (Data were obtained from triplicate experiments and expressed as the mean ± SD; *p < 0.05, **p < 0.01, ***p < 0.001 compared to the control or as indicated)


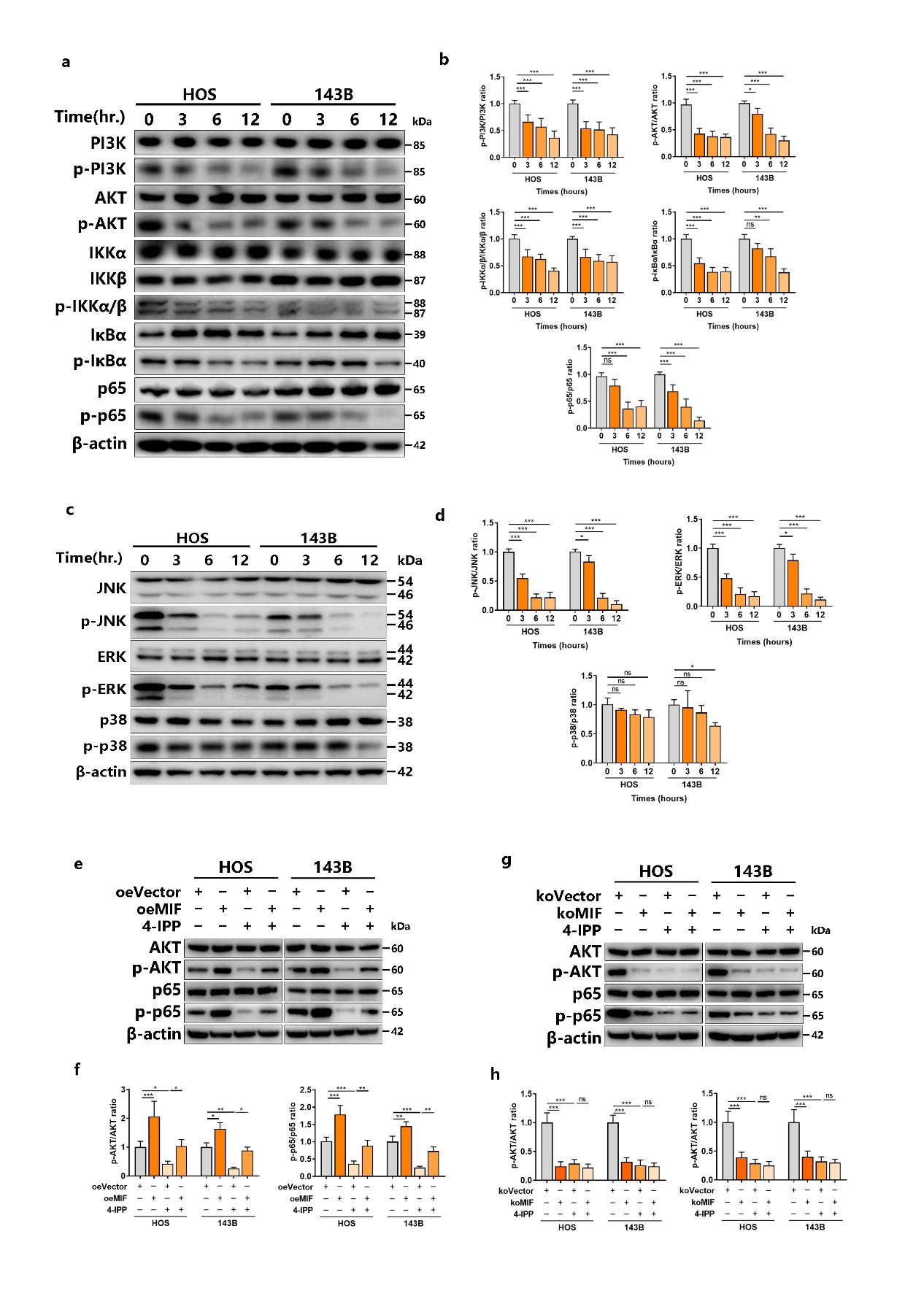


**Supplementary Figure 5.** **MIF inhibitor 4-IPP suppressed the PI3K/AKT/NF-κB and MAPK pathway.** (a) HOS and 143B cells received 3, 6, and 12 h of 4-IPP treatment, which was followed by western blotting-based analysis of the cell lysates. (b) The gray levels of phosphorylated p65, PI3K, IκBα, AKT, and IKKα/β were quantified and normalized to the total levels using ImageJ. (c) HOS and 143B cells received 3, 6, and 12 h of 4-IPP treatment, followed by Western blotting-based analysis of the cell lysates. (d) Gray levels of phosphorylated JNK, ERK and p38 were quantified and normalized to total JNK, ERK and p38 using ImageJ. (e) After stable transfection with the vector plasmid or MIF overexpression plasmid, HOS and 143B cells were treated with 4-IPP, followed by western blotting for AKT, p-AKT, p65, p-p65 and β-actin. (f) Gray levels of phosphorylated AKT and p65 were quantified and normalized to total AKT and p65 using ImageJ. (g) After stable transfection with the vector plasmid or MIF knockout plasmid, HOS and 143B cells were treated with 4-IPP, followed by tested by western blot for AKT, p-AKT, p65, p-p65 and β-actin. (h) Gray levels of phosphorylated AKT and p65 were quantified and normalized to total AKT and p65 using ImageJ. (Data were obtained from triplicate experiments and expressed as the mean ± SD; *p < 0.05, **p < 0.01, ***p < 0.001 compared to the control or as indicated)


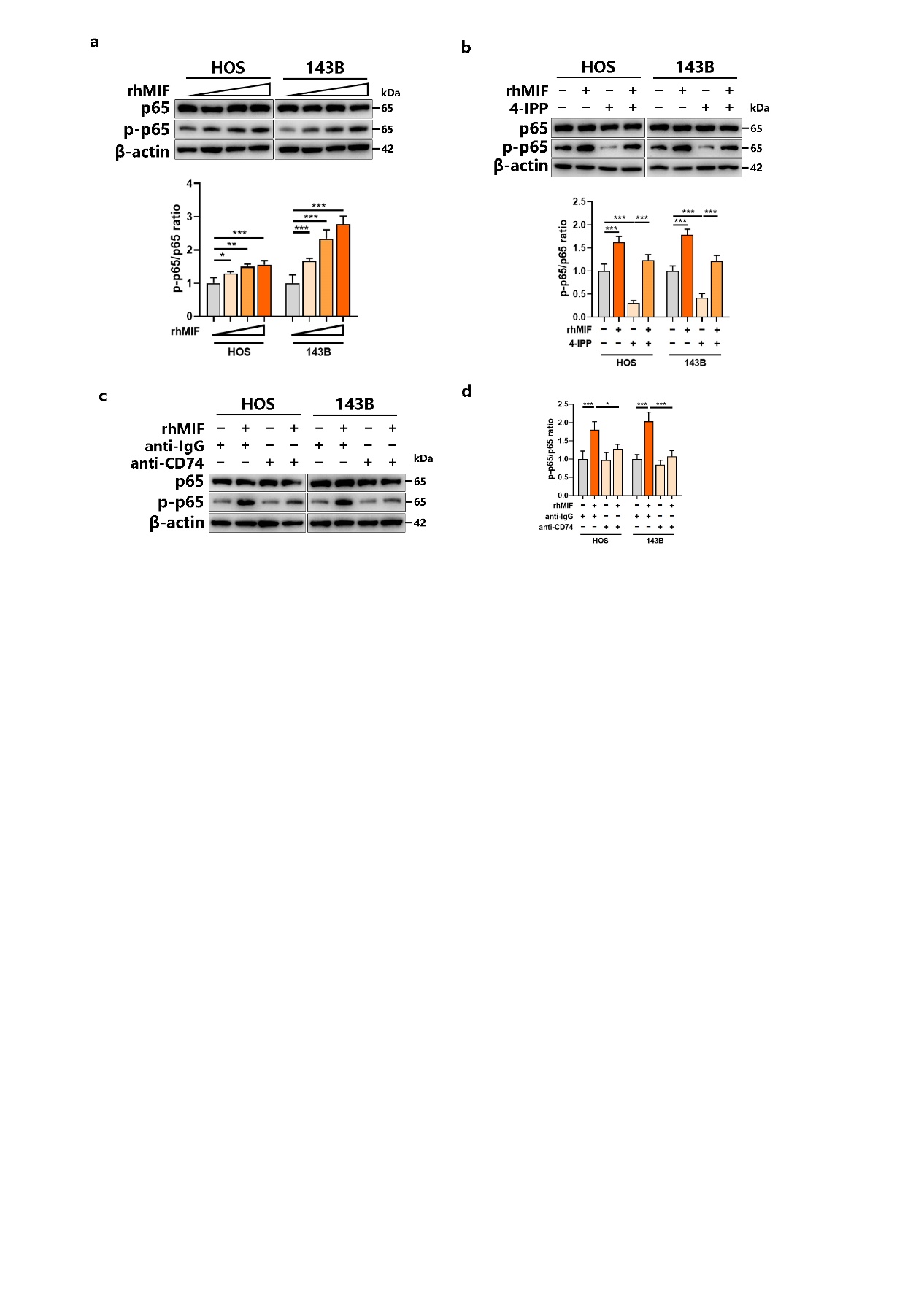


**Supplementary Figure 6. MIF activates the NF-κB pathway through CD74 receptors.** (a) HOS/143B cells received 1, 10, and 100ng/ml of rhMIF treatment, which was followed by western blotting-based analysis of the cell lysates (top). The gray levels of phosphorylated p65 were quantified and normalized to the total p65 levels using ImageJ (bottom). (b) HOS/143B cells were incubated with 40 μM 4-IPP or 100ng/ml of rhMIF for 24 h, followed by cell lysis (top). The gray levels of phosphorylated p65 were quantified and normalized to the total p65 levels using ImageJ (bottom). (c) HOS/143B cells were incubated with 40 μM 4-IPP, IgG antibody or CD74 antibody for 24, followed by cell lysis. (d) The gray levels of phosphorylated p65 were quantified and normalized to the total p65 levels using ImageJ.

**
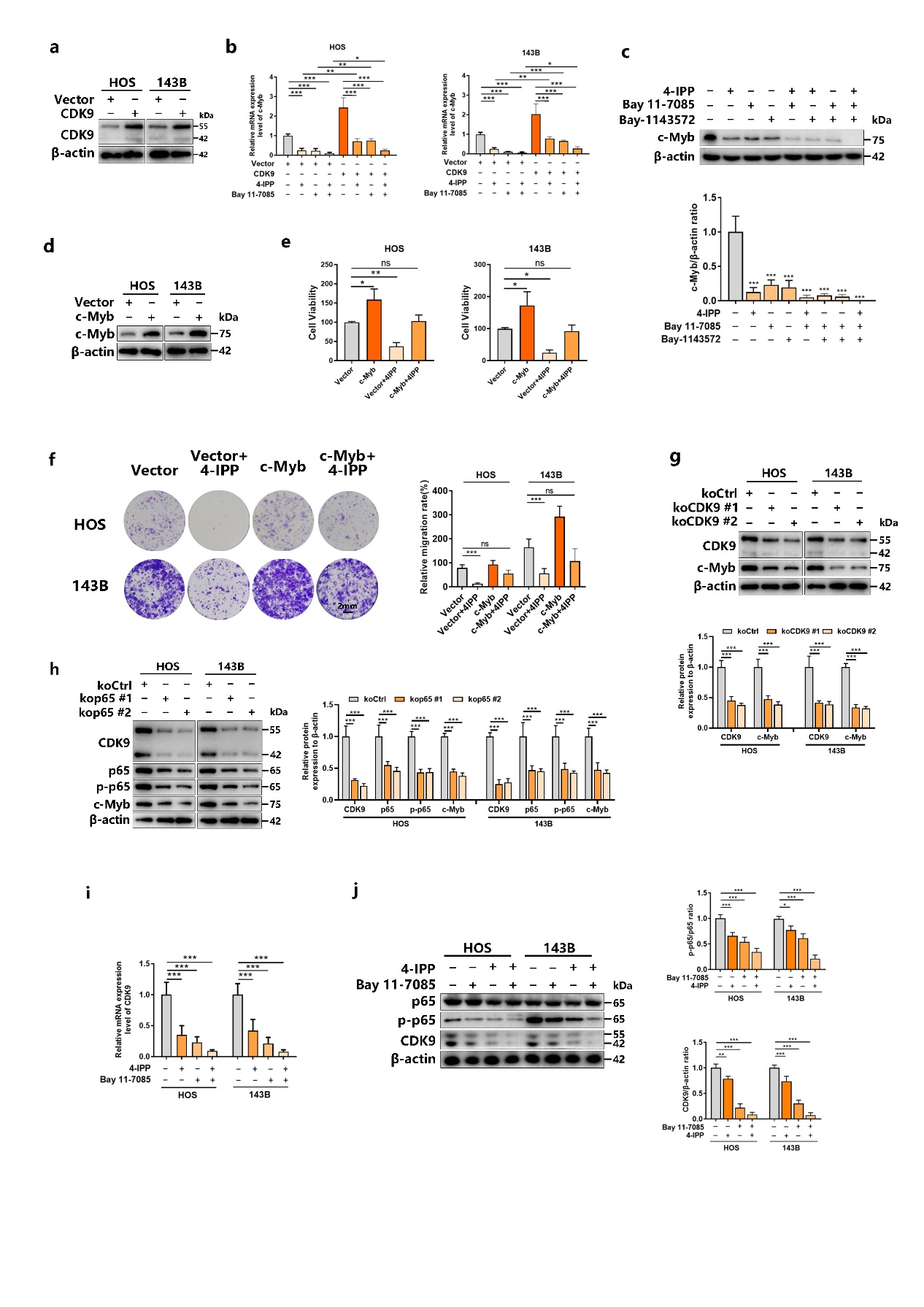
Supplementary Figure 7.** **c-Myb overexpression rescued the inhibition of 4-IPP in osteosarcoma.** (a) HOS and 143B cells were stably transfected with vector plasmid or CDK9 plasmid. Cells were lysed, and proteins were detected by western blotting. (b) HOS/143B cells were transfected with the CDK9 overexpression plasmid for 24 h, which was followed by 24 h of incubation with 40 μM 4-IPP or 10 μM Bay 11-7085 and qRT-PCR-based detection of the mRNA level of c-Myb. (c) HOS cells were incubated with 40 μM 4-IPP, 10 μM Bay 11-7085 or 10 μM Bay-1143572 for 24, followed by cell lysis. Proteins were then detected by western blotting (top) and c-Myb protein levels were quantified (bottom). (d) c-Myb protein expression detected by Western blot in HOS/143B cells transfected with the c-Myb overexpression plasmid. (e) HOS and 143B cells were stably transfected with vector plasmid or c-Myb overexpression plasmid and then treated with 4-IPP for 48 h. Viability was tested by CCK‐8 assays. (f) Effect of treatment with 4-IPP and overexpression of c-Myb on the colony-forming ability of osteosarcoma cells (left) was demonstrated by a colony formation assay and quantification of the number of colonies (right). (g) HOS/143B cells were transfected with the CDK9 knockout plasmid. Cells were lysed, and proteins were detected by western blotting (top). CDK9 and c-Myb protein levels were quantified (bottom). (h) After transfection with the vector plasmid or p65 knockout plasmid, HOS/143B cells were treated with 4-IPP, followed by tested by western blot for CDK9, c-Myb, p65, p-p65 and β-actin (left). Gray levels of phosphorylated AKT and p65 were quantified and normalized to total AKT and p65 using ImageJ (right). (i) HOS/143B cells received 24 h of treatment with 40 μM 4-IPP or 10 μM Bay 11-7085, which was followed by qRT-PCR-based detection of the mRNA level of CDK9. (j) HOS and 143B cells were treated with 40 μM 4-IPP or 10 μM Bay 11-7085 for 24 h and then lysed, and the indicated proteins were detected by western blotting (left) and quantified (right). (Data were obtained from triplicate experiments and expressed as the mean ± SD; *p < 0.05, **p < 0.01, ***p < 0.001 compared to the control or as indicated)


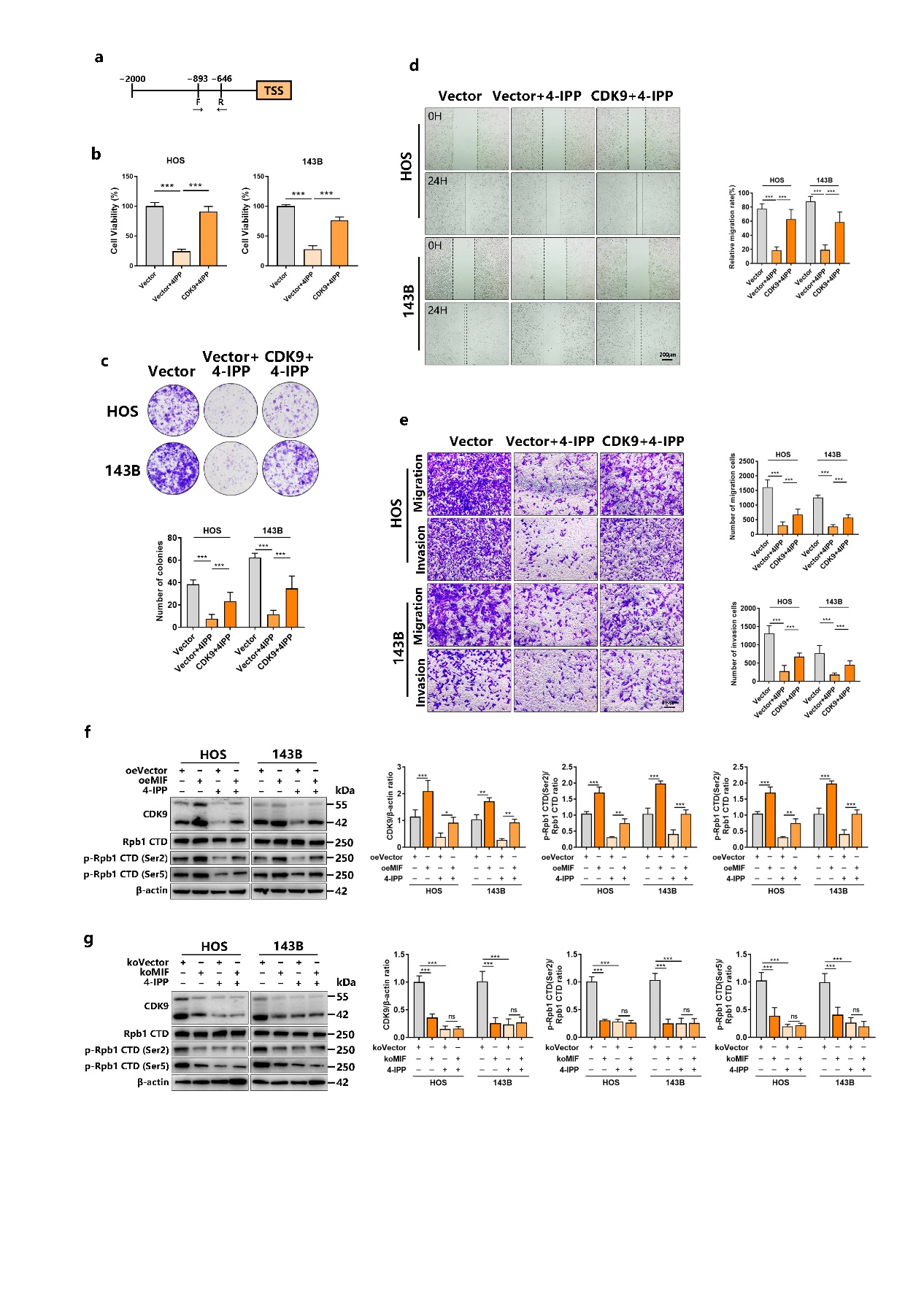


**Supplementary Figure 8.** **CDK9 overexpression rescues the inhibition of 4-IPP in osteosarcoma.** (a) Schematic of p65‐binding sites in the promoters of CDK9. (b) CDK9 protein expression detected by Western blot in 293T cells transfected with the CDK9 overexpression plasmid. (c) Effect of treatment with 4-IPP and overexpression of CDK9 on the colony-forming ability of osteosarcoma cells (top) was indicated by a colony formation assay and quantification of the number of colonies (bottom). (d) Reversion of migration ability by CDK9 overexpression was indicated by wound-healing assay. Scale bars, 200 μm. (e) Evaluation of cell migration and invasion was based on Transwell migration and Matrigel invasion assays (left), followed by quantification of the number of migrated or invaded cells (right). Scale bars, 100 μm. (f) HOS and 143B cells were stably transfected with vector plasmid or MIF overexpression plasmid and then treated with 4-IPP for 48 h, followed by cell lysis; proteins were then detected by western blotting (left) and protein levels were quantified (right). (g) HOS and 143B cells were stably transfected with vector plasmid or MIF knockout plasmid and then treated with 4-IPP for 48 h, followed by cell lysis; proteins were then detected by western blotting (left) and protein levels were quantified (right). (Data were obtained from triplicate experiments and expressed as the mean ± SD; *p < 0.05, **p < 0.01, ***p < 0.001 compared to the control or as indicated)


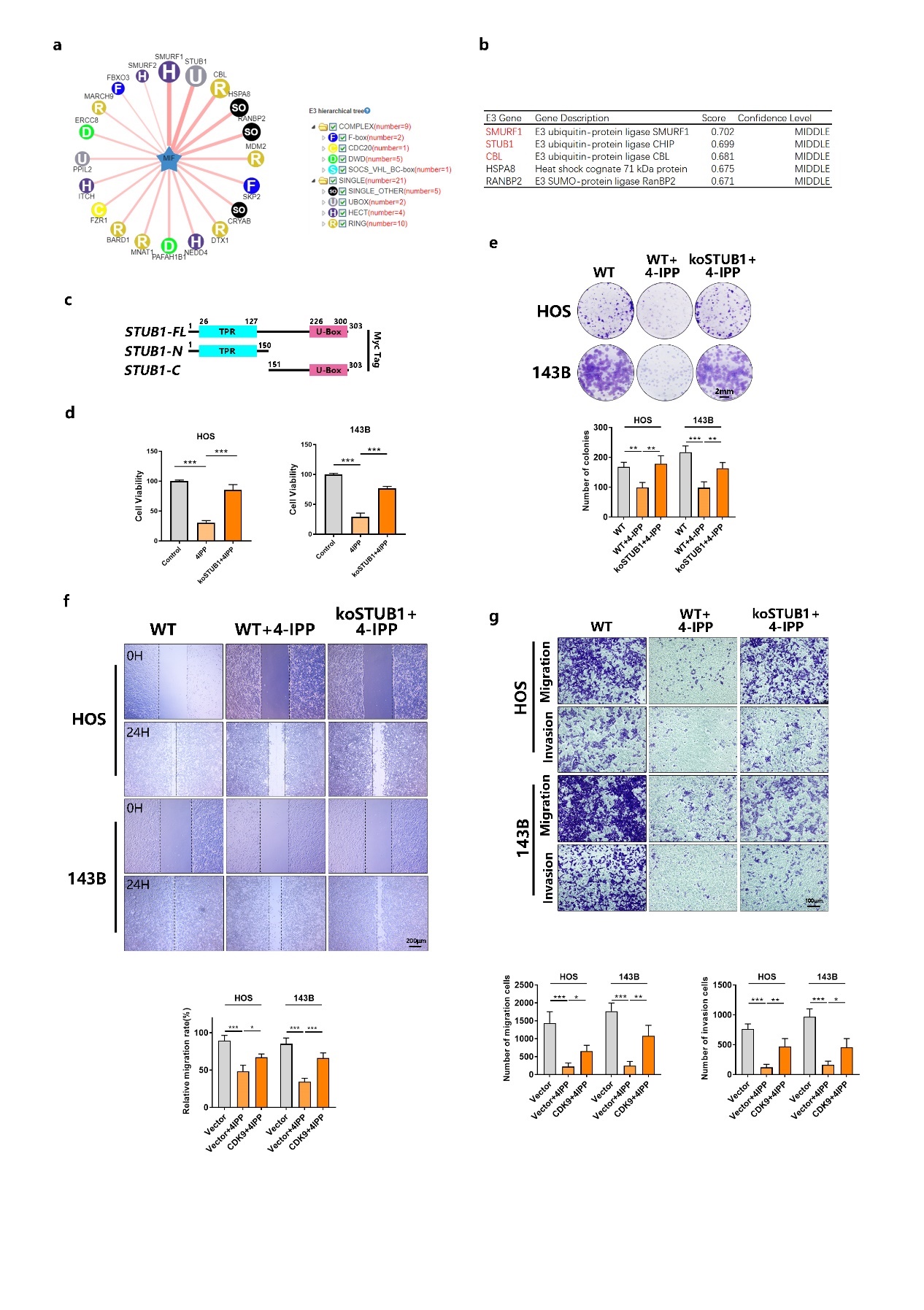


**Supplementary Figure 9.** **STUB1 overexpression rescues the inhibition of 4-IPP in osteosarcoma.** (a) Network view of the E3 hierarchical tree for MIF (upper right panel) and E3-MIF interactions (upper left panel). The E3 ligases for MIF were explored by UbiBrowser. Representative predicted E3 ligases surrounding MIF. The E3 type is reflected by node colors and characters. The confidence score was used to correct node size, edge shade and width. We depicted the predicted E3s and their position in the E3 family hierarchical tree, where the E3 family is denoted by texts in each circle (similar to “U,” “R,” and “H”). The number of corresponding predicted E3-MIF interactions is denoted by that in the bracket following each E3 family. (b) Information on the top five confidence levels of MIF E3 ligases. (c) Construction scheme of STUB1 protein truncation. (d) HOS and 143B cells were stably transfected with vector plasmid or STUB1 knockout plasmid and then treated with 4-IPP for 48 h. Viability was tested by CCK‐8 assays. (e) Effect of treatment with 4-IPP and knockout of STUB1 on the colony-forming ability of osteosarcoma cells (top) was demonstrated by a colony formation assay and quantification of the number of colonies (bottom). (f) Reversion of migration ability by STUB1 knockout was indicated by wound-healing assay. (g) Transwell migration and Matrigel invasion assays (top) were applied to evaluate cell migration and invasion. The number of migrated or invaded cells was quantified (bottom). Scale bars, 100 μm. (Data were obtained from triplicate experiments and expressed as the mean ± SD; *p < 0.05, **p < 0.01, ***p < 0.001 compared to the control or as indicated)


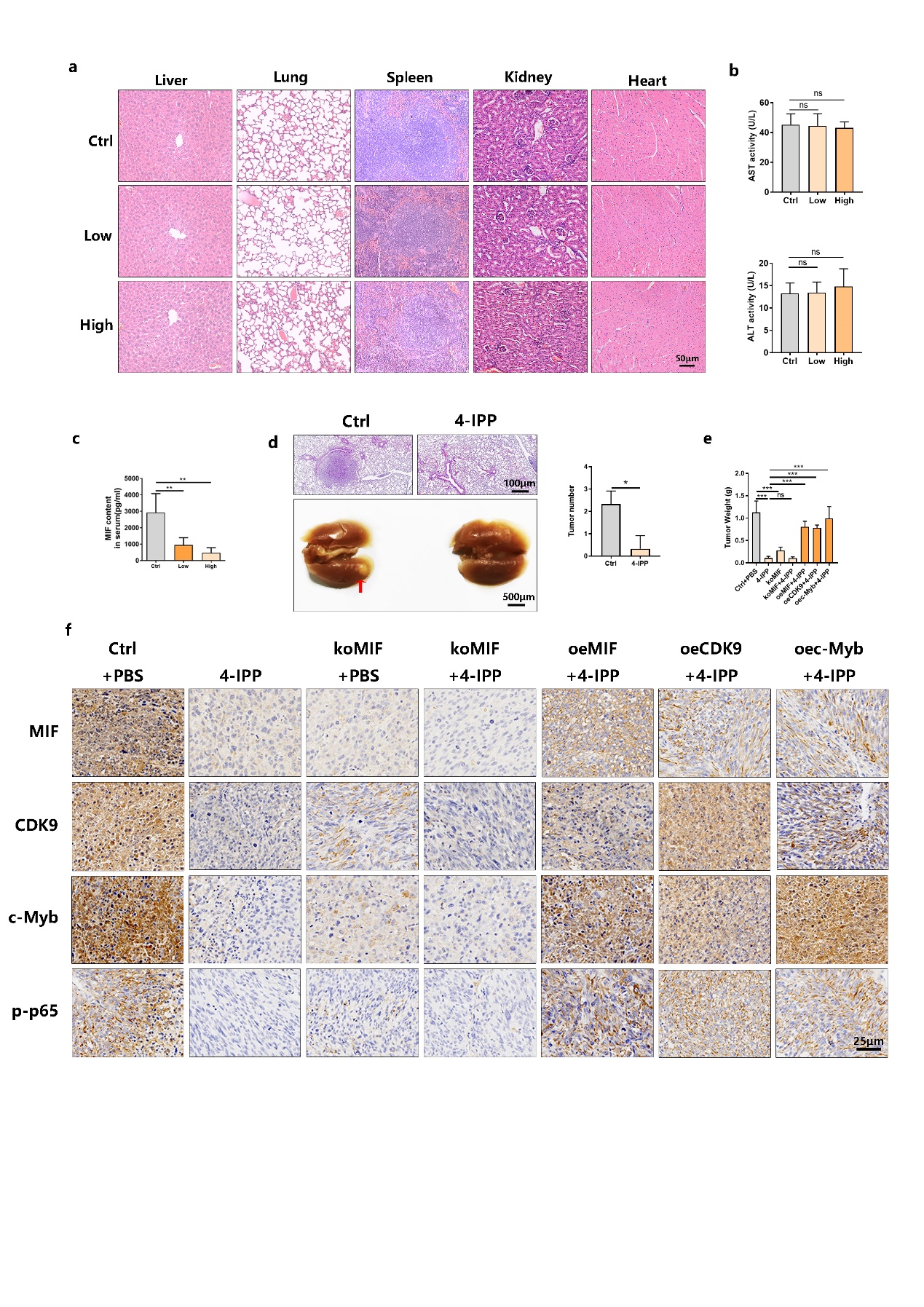


**Supplementary Figure 10.** **MIF degradation by 4-IPP suppresses osteosarcoma tumorigenesis and metastasis in vivo.** (a) H&E staining of the lung, spleen, kidney, heart and liver in different groups. Scale bar, 50 μm. (b) Effect of treatment with 4-IPP on the activity of AST (left) or ALT (right) was assessed (n=5). (c) Serum MIF content in different groups (n=5). (d) After injecting HOS cells into the tail veins of nude mice and sacrificing them (bottom), we separated the lungs and performed H&E staining (top) (n=3). Statistics on the number of tumors (right). (e) Tumor weight of the corresponding group (n=5). (f) Representative pictures of the indicated proteins detected by immunohistochemistry. Scale bar, 25 μm. (Data were obtained from triplicate experiments and expressed as the mean ± SD; *p < 0.05, **p < 0.01, ***p < 0.001 compared to the control or as indicated)


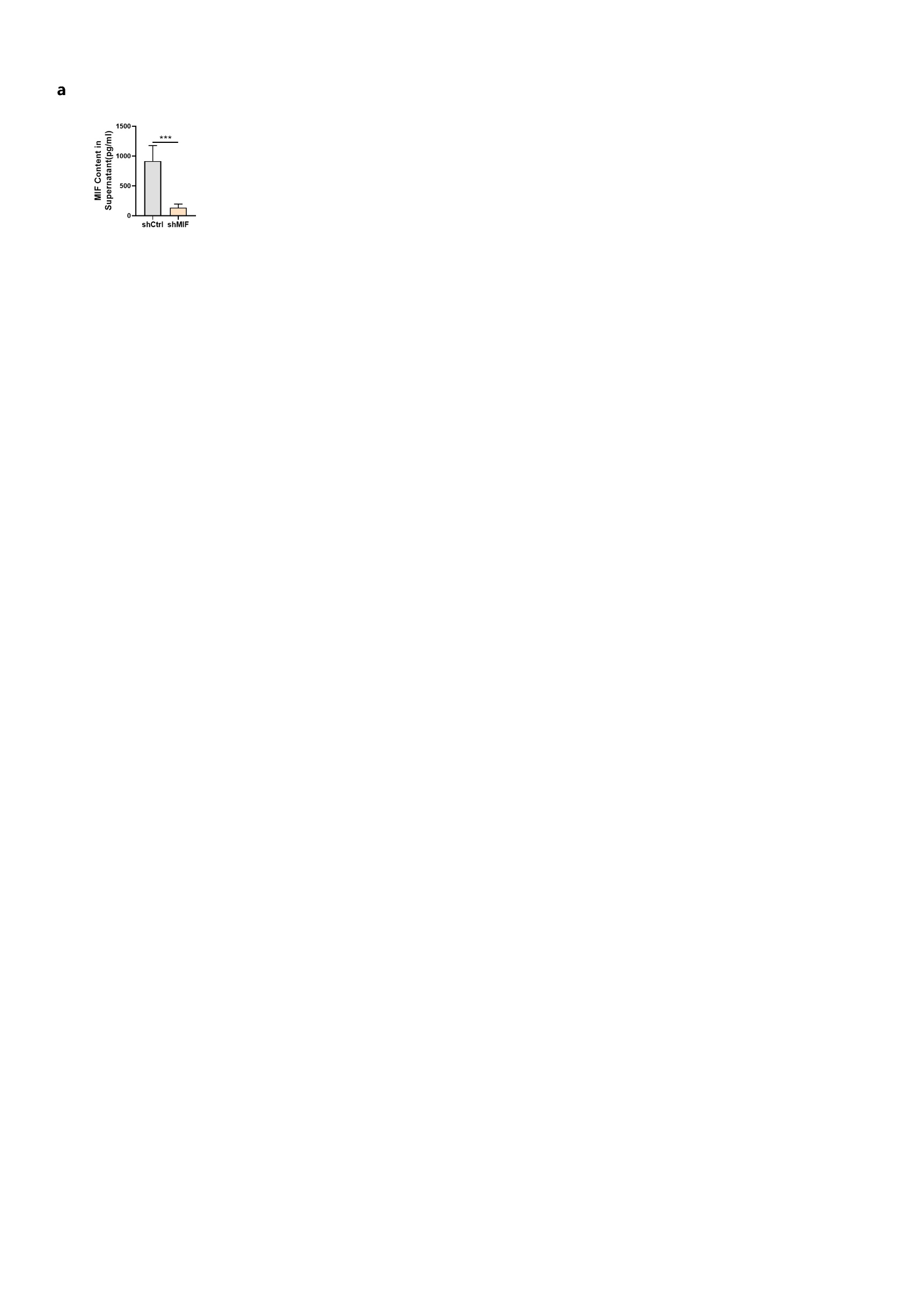


**Supplementary Figure 11. MIF content in cell supernatant**. (a) After MIF knockdown, the MIF content in the cell supernatant.

**Supplemental Experimental Procedures**

**Bone marrow macrophages (BMMs) isolation and in vitro osteoclast differentiation**

The isolation of BMMs was achieved by marrow flushing the long bones (femora and tibia) of 8-week-old mice littermates as previously described. In brief, 50 ng/ml receptor activator for nuclear factor-κ B ligand (RANKL) and 40 ng/ml macrophage colony-stimulating factor (M-CSF) were added to α-minimum essential medium (α-MEM) to culture the BMMs. Before mature multinucleated osteoclasts were formed, fresh media supplemented with RANKL and M-CSF was added to the BMM culture every other day for a total of 5 to 7 days.

**Cytotoxicity assay**

The cytotoxic effect of 4-IPP on osteosarcoma cells was examined using a Cell Counting Kit-8 (CCK-8) assay kit (Solarbio Life Sciences, Peking, China). Cells were seeded into 96-well plates in triplicate for 24 h for cell adhesion, and then they were treated with varying concentrations of 4-IPP for 24, 48 or 96 h. Subsequently, two h of cell incubation with CCK-8 reagent was performed. An ELX800 absorbance microplate reader was used to obtain absorbance at a wavelength of 450 nm.

**Colony formation assay**

Cells were seeded in a 12-well plate at a density of 600 cells/well, followed by 14 days of culturing. Then, 4% paraformaldehyde was used to fix cells for 20 minutes, and then, the cells were subjected to a 1-h staining process with a 0.5% crystal violet solution. Colony imaging was performed with an inverted light microscope (Zeiss, Primovert).

**Soft agar colony formation assay**

At a density of 10,000 cells/well, semisolid agar medium (0.4% agarose for culture medium with 0.6% agarose for the bottom layer in a 12-well plate) was used to seed the osteosarcoma cells. After incubation for 14 days, an inverted light microscope (Zeiss, Primovert) was used to obtain representative images of the cell colonies.

**Apoptosis analysis by flow cytometry**

An Annexin V-FITC/PI kit (BD Biosciences, San Diego, CA, USA) was adopted to determine apoptosis. Briefly, cells were trypsinized, washed with PBS, and stained with Annexin V-FITC/PI according to the manufacturer’s instructions. After incubation for 15 minutes, FlowJo software together with a flow cytometer (BD FACSCANTO II, BD Biosciences, San Jose, CA, USA) was used for cell analysis.

**Cell cycle analysis by flow cytometry.**

Cell cycle analysis was performed using a PI staining kit (BD Biosciences, San Diego, CA, USA). Briefly, the cells were harvested, washed with PBS, and then fixed in 75% cold ethanol at 4°C overnight. The cells were washed with PBS, incubated for 30 minutes with RNase A, and stained for 30 minutes with 500 μl PI at room temperature. Flow cytometry (BD FACSCANTO II, BD Biosciences) was applied for cell cycle analysis.

**Wound healing assay**

Osteosarcoma cells were cultured in a six-well plate, and then, the cell monolayer was scraped using a 200 μl pipette tip. At 0 and 24 h after injury, we captured representative images of cell migration and measured remodeling as the reduced distance across the induced injury area. Normalization to the 0 h control was then performed, and the results are presented as the relative migration rate.

**Transwell migration and invasion assays**

A Transwell chamber (BD Biosciences, Bedford, MA, USA) was used to perform Transwell migration and Matrigel-coated Transwell invasion assays. For the migration assay, a one-day incubation process was performed in 100 μl serum-free medium. In the Transwell chamber, 500 μl complete medium was placed in the lower chambers and 5 × 10^4^ cells were placed in the upper chambers. For the invasion assays, BD Matrigel™ Basement Membrane Matrix (BD Biosciences, Bedford, MA, USA) supplemented with 100 μl serum-free medium and 1 × 10^5^ cells was used to coat the upper chambers while 600 μl of complete medium was added to the lower chambers, and then a one-day incubation process was performed. An inverted light microscope (Zeiss, Primovert) was used to generate representative images, and the number of cells that migrated or invaded in no less than three random fields was counted.

**Gelatin zymography**

Experimental protocol for detecting MMP activity in conditioned medium using gelatin zymography was come from Abcam (<https://www.abcam.cn/protocols/gelatin-zymography-protocol>).

**Western blot analysis and co-immunoprecipitation (Co-IP)**

Laemmli sample buffer (1×), Bio-Rad, Berkeley, California, USA) was mixed with 2-mercaptoethanol to lyse cells. Total protein at equal amounts from different samples were separated by SDS-PAGE gels for 1.5 h at 80-120 V. Then, based on the indicated protein molecular weight, the proteins were transferred onto 0.22 μm polyvinylidene difluoride (PVDF) membranes (Thermo Fisher Scientific, Waltham, Massachusetts, USA) at 300 mA for 60-120 minutes. Then, the membrane was blocked for 1 h with 5% skimmed milk powder in TBST at room temperature, followed by an overnight incubation process with a specific primary antibody at 4°C. TBST was used to wash the membranes the next day, followed by incubation with an HRP-conjugated secondary antibody (Cell Signaling Technology, Boston, Massachusetts, USA). An electrochemiluminescence kit (Fudebio, Hangzhou, China) was used to detect each band. β-actin was used as the standard for normalization. For Co-IP, the cells were lysed in weak RIPA buffer for 20 minutes on ice and then incubated with Protein A/G MagBeads (YEASEN, Shanghai, China) and the indicated antibody according to the manufacturer’s protocols.

**Extraction of RNA and real-time quantitative PCR**

Gene expression levels were quantified through real-time quantitative PCR (qPCR). TRIzol (Invitrogen, Carlsbad, CA, USA) was applied to extract the RNA samples, and then a script RT reagent kit (TaKaRa) and SYBR Premix Ex Taq II (TaKaRa) were applied according to the manufacturer’s protocols. A Roche LightCycler® 480II PCR instrument (Basel, Switzerland) was used for reaction measurements. The 2−ΔΔCt method was adopted for data normalization to β-actin expression. The following Table lists the specific primers.

| Gene | Forward 5’ to 3’ | Reverse 5’ to 3’ |
| --- | --- | --- |
| *Human β-actin* | GGATTCCTATGTGGGCGACGA | GCGTACAGGGATAGCACAGC |
| *Human MIF* | CTCCACCTTCGCCTAAGAGC | TTCTCCCCACCAGAAGGTTG |
| *Human c-Jun* | GAGCTGGAGCGCCTGATAAT | CCCTCCTGCTCATCTGTCAC |
| *Human c-Fos* | CAGACTACGAGGCGTCATCC | TCTGCGGGTGAGTGGTAGTA |
| *Human c-Myc* | ACACTAACATCCCACGCTCTG | CGCATCCTTGTCCTGTGAGT |
| *Human c-Myb* | AAGTCTGGAAAGCGTCACTTG | ACATCTGTTCGATTCGGGAGATA |
| *Human KRAS* | TAGGCAAGAGTGCCTTGACG | CCCTCCCCAGTCCTCATGTA |
| *Human HRAS* | AAGTGTGTGCTCTCCTGACG | CACAAGGGAGGCTGCTGAC |
| *Human NRAS* | CCCGGCTGTGGTCCTAAATC | GCTTTTCCCAACACCACCTG |
| *Human CDK9* | GGTGAGGGAATTGGTGAGGG | CGTCCAAACTACTGCCACCT |
| *Human CD44* | CACACCCTCCCCTCATTCAC | TGGATGGCTGGTATGAGCTG |
| *Human CD74* | GGCAACATGACAGAGGACCA | CAGGATGTTGAAGACCGCCT |
| *Human CXCR2* | AGCTGAGAATATGCAGCCGT | GGTTGGGTGGTAGTCAGAGC |
| *Human CXCR4* | GTGACTTTGAAACCCTCAGCG | TGGGCTAAGGGCACAAGAGA |
| *Human CXCR7* | ATTTGATTGCCCGCCTCAGA | GACGCTTTTGTTGGGCATGT |
| *Mouse β-actin* | ACAGCAGTTGGTTGGAGCAA | ACGCGACCATCCTCCTCTTA |
| *Mouse NFATc1* | TCCACCCACTTCTGACTTCC | CTTCGCCCACTGATACGAG |
| *Mouse TRAP* | CCATTGTTAGCCACATACGG | CACTCAGCACATAGCCCACA |
| *Mouse c-Fos* | GTTCGTGAAACACACCAGGC | GGCCTTGACTCACATGCTCT |
| *Mouse CTSK* | TCCGCAATCCTTACCGAATA | AACTTGAACACCCACATCCTG |

**Luciferase reporter assay**

We cotransfected the luciferase reporters pGL and pRL-TK into cells with Lipofectamine 3000. The Dual-Luciferase Reporter Assay System (Beyotime, Shanghai, China) and Modulus Single Tube Luminometer (Turner Biosystems, Sunnyvale, California, USA) were applied to measure P65 transcriptional activity and chemiluminescence, respectively.

**Chromatin immunoprecipitation assays**

ChIP assays were performed using a SimpleChIP® Plus Enzymatic Chromatin IP Kit (Cell Signaling Technology, Boston, Massachusetts, USA) according to the manufacturer’s instructions. In detail, the cells were fixed with 1% formaldehyde and then quenched with 125 mM glycine, which was followed by sonication of the nuclear extract. After 1 h of preclearing with normal IgG, an incubator was used to immunoprecipitate the sonicated cell lysate and indicated antibody overnight at 4°C. Protein A/G magnetic beads were added the next day, followed by another 2 h of incubation of the cell lysate. After the buffer washing process, we performed reverse cross-linking by eluting the chromatin from the protein/DNA complex, and then digestion was performed with proteinase K and RNase A overnight at 65°C. An AxyPrep PCR cleanup kit (Axygen) was used to purify the freed DNA, and SYGR Green master mix was applied for a quantitative PCR analysis. Supplementary Table 1 lists all the sequences of primers.

**Tail vein lung metastasis model,** **subcutaneous and orthotopic xenograft tumor model**

Male nude mice at four weeks old were used as in vivo tumor models. For the tail vein lung metastasis model, we injected the HOS cell lines (equivalent volume and density) into the tail vein of each nude mouse after suspending them in sterile PBS. One week later, we randomly assigned the mice to three groups, which were subjected to intraperitoneal injections of PBS,5mg/kg or 20 mg/kg 4-IPP. After 3 weeks, the mice were sacrificed and their tumors were fixed in 4% paraformaldehyde. For subcutaneous and orthotopic xenograft tumor models, a total of 5 × 10^6^ HOS cells were injected on one side of the lower dorsal flank (n=5 per group) or into the cavity of the tibia (n=3 per group). The tumor volume calculation formula was as follows: volume (mm^3^)=ab^2^/2. One week later, we randomly assigned mice into three groups, which were subjected to intraperitoneal injections of PBS, 5 mg/kg 4-IPP or 20 mg/kg 4-IPP. The mice were sacrificed 30 days after the injection, and the tumors were fixed in 4% paraformaldehyde.

**MicroCT Imaging**After fixation in 4% PFA, a SkyScan 1072 micro-CT system (Brook, Belgium) was used to scan the tibia with a current of 80 μA, equidistant pixels at 9 μm, and a voltage of 70 kV. All images were analyzed by SkyScan CTAn software (Bruck Corporation) after reconstruction by the SkyScan NRecon program (Bruck Corporation). We quantitatively and qualitatively analyzed the bone parameters in a square area of interest and set 0.5 mm below the growth plate. The analyzed parameters included the average trabecular number, total porosity, bone volume/tissue volume percentage (BV/TV, %), average trabecular thickness (Tb.Th), and average trabecular spacing (Tb.Sp).

**Immunofluorescence staining**

Immunofluorescence was used to assess protein expression levels and protein colocalization. The cells were fixed with paraformaldehyde and permeabilized with 0.25% Triton X-100, followed by 2 h of incubation with the antibody at 4°C. Then, a fluorescein-conjugated secondary antibody was added to the cells, which were incubated for 1 h at 4°C in the dark, followed by a counterstaining process of 15 minutes with DAPI at room temperature in the dark to stain the nuclei. A Nikon A1 confocal microscope (Nikon, Japan) with a digital camera was used to capture fluorescence images. The percentage of p65-positive nuclear cells was calculated using ImagePro Plus (Media Cybernetics, Maryland, USA).

**Key Resources Table**

| PEAGENT or RESOURCE | SOURCE | IDENTIFIER |
| --- | --- | --- |
| Antibodies | | |
| MIF | Abcam | Cat # ab175189 |
| β-actin | Beyotime | Cat # AA128 |
| Ubiquitin | CST | Cat # 3936 |
| Bax | CST | Cat # 5023 |
| Bcl-2 | CST | Cat # 15071 |
| Cleaved-caspase 3 | CST | Cat # 9664 |
| Cleaved-PARP | CST | Cat # 5625 |
| N-cadherin | CST | Cat # 13116 |
| E-cadherini | CST | Cat # 14472 |
| Vimentin | CST | Cat # 5741 |
| MMP2 | Proteintech | Cat # 10373-2-AP |
| MMP9 | Proteintech | Cat # 10375-2-AP |
| PI3K | CST | Cat # 4257 |
| p-PI3K (Tyr458) | CST | Cat # 4228 |
| AKT | CST | Cat # 4691 |
| p-AKT (Ser473) | CST | Cat # 4060 |
| IKKα | Abcam | Cat # ab32041 |
| IKKβ | Abcam | Cat # ab124957 |
| p-IKKα/β (Ser176/180) | CST | Cat # 2697 |
| IκBα | CST | Cat # 4814 |
| p-IκBα (Ser32) | CST | Cat # 2859 |
| p65 | CST | Cat # 8242 |
| p-p65 (Ser536) | CST | Cat # 3033 |
| Histone H3 | CST | Cat # 4499 |
| c-Myb | Santa Cruz | Cat # sc-74512 |
| CDK9 | CST | Cat # 2316 |
| Rbp1 CTD | CST | Cat # 2629 |
| p-Rbp1 CTD (Ser2) | CST | Cat # 13499 |
| p-Rbp1 CTD (Ser5) | CST | Cat # 13523 |
| Flag | CST | Cat # 14793 |
| Myc | CST | Cat # 2276 |
| JNK | CST | Cat # 9252 |
| p-JNK (Thr183/Tyr185) | CST | Cat # 4668 |
| ERK | CST | Cat # 4695 |
| p-ERK (Thr202/Tyr204) | CST | Cat # 4370 |
| p38 | CST | Cat # 8690 |
| p-p38 (Thr180/Tyr182) | CST | Cat # 4511 |
| normal mouse IgG | Santa Cruz | Cat # sc-2025 |
| Anti-mouse IgG, HRP-linked Antibody | CST | Cat # 7076 |
| Anti-rabbit IgG, HRP-linked Antibody | CST | Cat # 7074 |
| Chemicals, Peptides, and Recombinant Proteins | | |
| 4-IPP | Tocris | 3429 |
| Bay 11-7085 | MCE | HY-10257 |
| Bay-1143572 | Selleck | S8727 |
| Recombinant MIF protein | R&D Systems | 289-MF |
| Critical Commercial Assays | | |
| MIF ELISA Kit | Cusabio | CSB-E08330h |
| Annexin V-FITC/PI Apoptosis Detection Kit | BD Biosciences | 556547 |
| PI/RNase Staining Buffer | BD Biosciences | 550825 |
| SimpleChIP® Enzymatic Chromatin IP Kit | CST | 9002 |
| Experimental Models: Cell Lines | | |
| hFOB1.19 | ATCC | CRL-11372 |
| MG63 | ATCC | CRL-1427TM |
| HOS | ATCC | CRL-1543 |
| 143B | ATCC | CRL-8303 |
| SJSA-1 | ATCC | CRL-2098 |
| U2OS | ATCC | HTB96TM |
| Recombinant DNA | | |
| lentiCRISPR v2 | Addgene | 49535 |
| pCMV6-Entry | OriGene | PS100001 |
| Flag-MIF | This paper | N/A |
| Flag-SMURF1 | This paper | N/A |
| Flag-STUB1 | This paper | N/A |
| Flag-CBL | This paper | N/A |
| Myc-STUB1-FL | This paper | N/A |
| Myc-STUB1-N | This paper | N/A |
| Myc-STUB1-C | This paper | N/A |
| Flag-c-Myb | This paper | N/A |
| Flag-CDK9 | This paper | N/A |
| Oligonucleotide | | |
| MIF shRNA: ACATCAACTATTACGACAT | This paper | N/A |
| MIF sgRNA: TCAGGCACGTAGCTCAGCGG | This paper | N/A |
| STUB1 sgRNA: CTCTTCGGAACCCGCCTCGG | This paper | N/A |
| CDK9 sgRNA #1: CTCACCGTATAACCGCTGCA | PMID: 30209133 | N/A |
| CDK9 sgRNA #2: CACCGGCTCGCAGAAGTCGAACACC | PMID: 34004147 | N/A |
| p65 sgRNA #1: AGCGCCCCTCGCACTTGTAG | PMID: 31105691 | N/A |
| p65 sgRNA #2: CAAGTGCGAGGGGCGCTCCG | PMID: 31105691 | N/A |
| Software and Algorithms | | |
| GraphPad Prism 8 | GraphPad Software | N/A |
| ImageJ v1.50 |  | https://imagej.nih.gov/ij/download.html |
| Flowjo10 | Core Facilities Zhejiang University School of Medicine | N/A |
